# ﻿Six new species of the spider genus *Clubiona* Latreille, 1804 (Araneae, Clubionidae) from subtropical forests of Sichuan Province, China

**DOI:** 10.3897/zookeys.1248.153967

**Published:** 2025-08-04

**Authors:** Jianshuang Zhang, Yuanqian Xing, Hao Yu, Shuqiang Li

**Affiliations:** 1 The State Key Laboratory of Southwest Karst Mountain Biodiversity Conservation of Forestry Administration, School of Life Sciences, Guizhou Normal University, Guiyang, Guizhou 550025, China Guizhou Normal University Guiyang China; 2 College of Life Sciences, Anhui Normal University, Wuhu, Anhui 241000, China Anhui Normal University Beijing China

**Keywords:** Asia, DNA barcoding, morphology, sac spiders, species group, taxonomy

## Abstract

Six new species, belonging to three species groups of *Clubiona* Latreille, 1804 are described from both males and females: *C.huntianling* Yu & Li, **sp. nov.**, *C.rouqiu* Yu & Li, **sp. nov.** and *C.yinyangjian* Yu & Li, **sp. nov.** from the *corticalis* group; *C.huojianqiang* Yu & Li, **sp. nov.** and *C.qiankunquan* Yu & Li, **sp. nov.** from the *trivialis* group; *C.nezha* Yu & Li, **sp. nov.** from the *zilla* group. These species are currently known to occur only in subtropical forests, Sichuan, China. The DNA barcodes of all species were obtained for species delimitation, matching of sexes and future use.

## ﻿Introduction

*Clubiona* Latreille, 1804 is the most species-rich genus within the Clubionidae Simon, 1878, with 530 currently recognized extant species. It is widely distributed across various regions and countries, except for the Antarctic and Neotropics (WSC 2025). To date, 178 *Clubiona* species have been recorded in China, accounting for approximately one-third of the global diversity of the genus, making China the country with the highest species richness of *Clubiona* (WSC 2025). Nevertheless, the species diversity of this genus in China remains incompletely documented, as numerous new species have been described in recent years (Xin et al. 2020; Zhang and Yu 2020; Gan and Wang 2020; Zeng et al. 2021; Zhang et al. 2021, 2022; Zhong et al. 2022; Li et al. 2023; Wu et al. 2023; Guo et al. 2025; WSC 2025).

**Figure 1.**
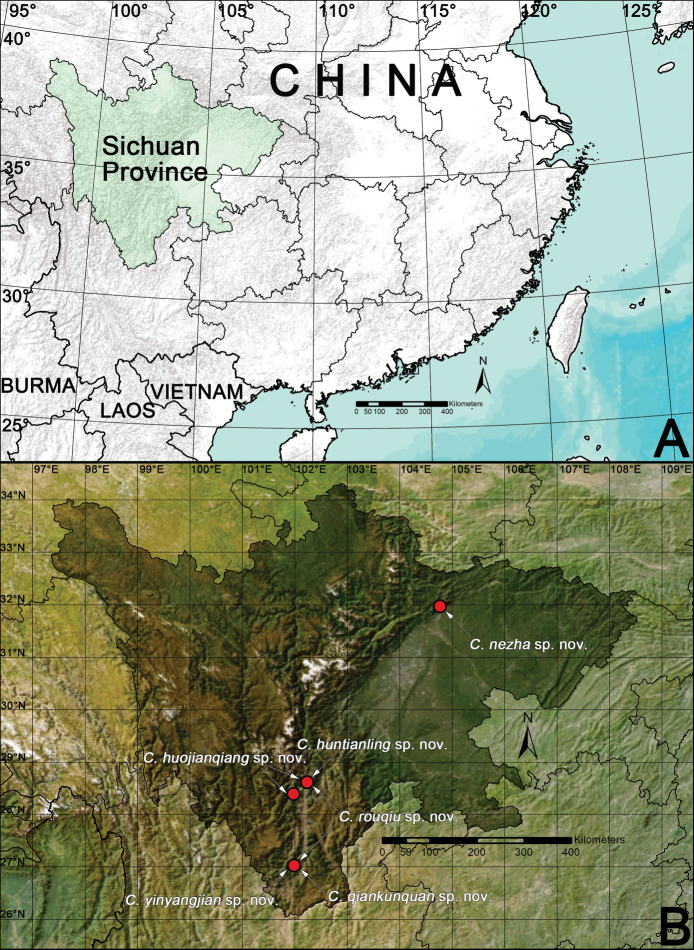
A. Locality of Sichuan Province; B. distribution of the new *Clubiona* species from Sichuan.

Recently, an intensive expedition in Sichuan Province, Southwest China (Fig. 1A) was carried out by the colleagues of the Chinese Academy of Sciences and Shenyang Normal University. In this paper, six new species of *Clubiona* brought to light by those expeditions are described from three species groups: *corticalis*, *trivialis*, and *zilla* (Fig. 1B).

## ﻿Materials and methods

All specimens were collected by canopy fogging. Specimens were preserved in 95% ethanol. All type specimens are deposited in the Institute of Zoology, Chinese Academy of Sciences (**IZCAS**) in Beijing, China (curator Jun Chen).

Specimens were examined using a LEICA M205C and an Olympus SZX7 stereomicroscope. Further details were studied under an Olympus CX41 compound microscope. Male and female copulatory organs were examined and illustrated after dissection. Left male palps were illustrated. Epigynes were removed and cleared in lactic acid or warm 10% potassium hydroxide (KOH) solution. Some vulvae were imaged after being embedded in Arabic gum. Images were captured with a Canon EOS 70D digital camera mounted on an Olympus CX41 compound microscope and assembled using Helicon Focus 6.80 image stacking software. All measurements were obtained using an Olympus SZX7 stereomicroscope and are given in millimetres. Eye diameters are taken from the widest distance. The total body length does not include chelicerae or spinnerets. Leg lengths are given as total length (femur, patella + tibia, metatarsus, tarsus). Terminology in the text and figure legends follows Yu and Li (2019a, b), Yu et al. (2017), and Zhang et al. (2018, 2021). The distribution map was generated with ArcGIS 10.5 (ESRI Inc).

Abbreviations used in the text and figures are as follows:

**A** atrium

**AAM** atrial anterior margin

**AER** anterior eye row

**ALE** anterior lateral eyes

**AME** anterior median eyes

**AME–ALE** distance between AME and ALE

**AME–AME** distance between AMEs

**APM** atrial posterior margin

**AS** anterior part of spermatheca

**BS** bursa

**C** conductor

**CBE** cymbial base extension

**CD** copulatory duct

**CO** copulatory opening

**Cy** cymbium

**DTA** dorsal tibial apophysis

**EB** embolic base

**EBP** embolic base process

**Em** embolus

**EP** embolic projection

**FD** fertilisation duct

**H** hood

**MOQ** median ocular quadrangle

**MOQA**MOQ anterior width

**MOQL** length of MOQ

**MOQP**MOQ posterior width

**MS** median part of spermatheca

**PA** patellar apophysis

**PER** posterior eye row

**PLE** posterior lateral eyes

**PME** posterior median eyes

**PME–PLE** distance between PME and PLE

**PME–PME** distance between PMEs

**PS** posterior part of spermatheca

**RTA** retrolateral tibial apophysis

**SD** sperm duct

**SG** spermathecal gland

**Sp** spermatheca

**Sp1** primary spermatheca

**Sp2** secondary spermatheca

**St** subtegulum

**Te** tegulum

**TG** tegular groove

**TH** tegular hump

**VTA** ventral tibial apophysis

DNA barcodes were obtained for species delimitation and matching of sexes. A partial fragment of the mitochondrial cytochrome oxidase subunit I (CO1) gene was amplified and sequenced for 21 specimens using the primers LCO1490 (5’-GGTCAACAAATCATAAAGATATTG-3’) and HCO2198 (5’-TAAACTTCAGGGTGACCAAAAAAT-3’). For additional information on extraction, amplification, and sequencing procedures, see Malumbres-Olarte and Vink (2012).

Sequences were trimmed to 631 bp. All sequences were confirmed using BLAST and are deposited in GenBank. The codes and GenBank accession numbers of voucher specimens are provided as follows: *C.huntianling* sp. nov.: YHCLU0389, ♂, GenBank PV808722; YHCLU0390, ♀, GenBank PV808723. *C.rouqiu* sp. nov.: YHCLU0393, ♂, GenBank PV808726; YHCLU0382, ♀, GenBank PV808719. *C.yinyangjian* sp. nov.: YHCLU0395, ♂, GenBank PV808727; YHCLU0396, ♀, GenBank PV808728. *C.huojianqiang* sp. nov.: YHCLU0402, ♀, GenBank PV808729. *C.qiankunquan* sp. nov.: YHCLU0385, ♂, GenBank PV808720; YHCLU0386, ♀, GenBank PV808721. *C.nezha* sp. nov.: YHCLU0391, ♂, GenBank PV808724; YHCLU0392, ♀, GenBank PV808725.

## ﻿Taxonomic account

### ﻿Family Clubionidae Wagner, 1887

#### ﻿Genus Clubiona

Taxon classificationAnimaliaAraneaeClubionidae

Latreille, 1804

FCA4FEEE-9A57-5A0E-88BF-5CFF10ED7E61

##### Comments.

Although several major taxonomic studies on a regional scale have been conducted, e.g., Simon (1932) for the French species, Lohmander (1944) and Almquist (2007) for the Swedish species, Wiehle (1965) for the German species, Wunderlich (2011) for the European species, Edwards (1958) for the North American species, Dondale and Redner (1982) for the Canadian and Alaskan species, Mikhailov (1990, 1991, 1995, 2002, 2012) for the Palaearctic species, and Deeleman-Reinhold (2001) for the Southeast Asian species, the debate on the genus’s limits and internal structure of this genus still remains open (Zhang et al. 2021).

There are 14 genus group names that are currently considered junior synonyms of *Clubiona* (see the list in WSC (2025), Marusik and Omelko (2018) and Zhang et al. (2021)). In addition, at least ten subgeneric names and 20 species group names have been proposed for subdivisions within the genus (Lohmander 1944; Dondale and Redner 1982; Mikhailov 1990, 1991, 1995, 2002; Deeleman-Reinhold 2001). However, most of these generic and subgeneric classifications were later suppressed by Mikhailov (1990, 1991, 1995, 2002, 2012) and Deeleman-Reinhold (2001). Currently, only a dozen species group names remain in use for the taxonomy of the genus. While there is no consensus on the delimitation of most species groups, some are characterized by a distinct set of morphological traits and a relatively stable species composition, making them widely accepted among taxonomists. At least 18 species groups are frequently discussed or referenced in recent literature (Table 1).

**Table 1.** Checklist of species group names of *Clubiona* that were frequently used in recent publications.

Current species group names	Equivalent genus group name (genus, subgenus)	Note
*C.abboti* grp	–	–
*C.brevipes* grp	*Breviclubiona* Wunderlich, 2011	–
*C.caerulescens* grp	*Gauroclubiona* Lohmander, 1944	–
*C.comta* grp	*Hyloclubiona* Lohmander, 1944	includs *C.genevensis* grp which was used in Almquist (2007)
*C.corticalis* grp	*Atalia* Thorell, 1887; *Paraclubiona* Lohmander, 1944	–
*C.filicat*a grp	*Tolophus* Thorell, 1891; *Japoniona* Mikhailov, 1990	corresponds to *C.japonica* grp which was used in Deeleman-Reinhold (2001) and Yu et al. (2017)
*C.lutescens* grp	*Heteroclubiona* Lohmander, 1944	corresponds to *C.terrestris* grp and *C.japonicola* grp which were used in Almquist (2007) and Zhang et al. (2021), respectively
*C.marmorata* grp	*Marmorclubiona* Wunderlich, 2011	–
*C.maritima* grp	–	–
*C.milingae* grp	–	corresponds to *C.apiculata* grp which was used in Dankittipakul and Singtripop (2014) and Yu and Li (2019a)
*C.obesa* grp	–	belongs to *Clubiona* s. str.
*C.pahilistapyasea* grp	–	–
*C.pallidula* grp	–	belongs to *Clubiona* s. str.
*C.reclusa* grp	*Euryclubiona* Lohmander, 1944	–
*C.similis* grp	*Epiclubiona* Lohmander, 1944	corresponds to *C.frisia* grp which was used in Almquist (2007)
*C.ternatensis* grp	*Hirtia* Thorell, 1881	corresponds to *C.hystrix* grp which was used in Deeleman-Reinhold (2001) and Yu and Li (2019a, b)
*C.trivialis* grp	*Microclubiona* Lohmander, 1944	–
*C.zilla* grp	*Anaclubiona* Ono, 2010	–

Due to the highly diverse copulatory structures in both sexes, *Clubiona* sensu lato has long been considered paraphyletic and is likely to be split in the future (Wunderlich 2011; Marusik and Omelko 2018; Zhang et al. 2021). However, we concur with Mikhailov (2012) that a comprehensive, large-scale revision of the genus is necessary. Therefore, following the classifications of Mikhailov (2012) and the WSC (2025), we provisionally assign all species treated in this study to *Clubiona* sensu lato.

### ﻿*Clubionacorticalis* group

**Comments.** At least two generic names are available for the *corticalis* group: *Atalia* Thorell, 1887 (type species: *A.concinna*, currently considered as a member of the *corticalis* group) and *Paraclubiona* Lohmander, 1944 (type species: *C.corticalis*) (Zhang et al. 2018, 2021). Both taxa are currently recognized as junior synonyms of *Clubiona* (Wu et al. 2015; WSC 2025). The most recent global checklist of *C.corticalis* group encompasses 81 species (Zhang et al. 2021). Since then, nine additional species have been successively assigned to this group: *C.xianning* Zhong & Yu, 2022, *C.bidactylina* Wu, Chen & Zhang, 2023, *C.camela* Wu, Chen & Zhang, 2023, *C.subhuiming* Wu, Chen & Zhang, 2023, *C.tianpingshan* L. F. Li, Liu, B. Li & Peng, 2023, *C.longyangensis* Guo, Li & Zhang, 2025, *C.multiprocessa* Guo, Li & Zhang, 2025 from China, and *C.dorni* Sarkar, Quasin & Siliwal, 2023 and *C.uniyali* from India (Zhong et al. 2022; Wu et al. 2023; Li et al. 2023; Sarkar et al. 2023; Guo et al. 2025).

The *corticalis* group is among the most species-rich assemblages within *Clubiona* and can be further divided into at least six or seven distinct lineages based on morphological features and molecular data (pers. obs.). Some of these lineages appear to differ significantly from one another and show little or no close relationship to *C.corticalis* itself, suggesting that the current *corticalis* group is likely polyphyletic. As such, the group cannot be formally diagnosed at present due to the absence of clear synapomorphies. However, a comprehensive revision of *Clubiona* sensu lato and the *corticalis* group is beyond the scope of the present study. The three new species described in this paper bear resemblance to certain species currently placed within the *corticalis* group. Therefore, in the absence of a better alternative, we tentatively assign them to the *corticalis* group in the present work.

#### ﻿ Clubiona huntianling

Taxon classificationAnimaliaAraneaeClubionidae

Yu & Li sp. nov.

A56793E6-4BF4-521D-80E9-736E121994F0

https://zoobank.org/7927587B-E616-42D2-8961-B16AF361DFCB

Figs 1 , 2 , 3

##### Type material.

***Holotype***: China • ♂ (IZCAS-Ar 45529, YHCLU0389); Sichuan Prov., Liangshan Pref., Mianning Co., Yihai Town, Damawu Vill.; 28.61°N, 102.24°E, ca 2213 m; 9.VI.2024; X. Zhang et al. leg. ***Paratype***: China • 1 ♀ (IZCAS-Ar 45530, YHCLU0390); same data as for holotype.

##### Diagnosis.

**Figure 2.**
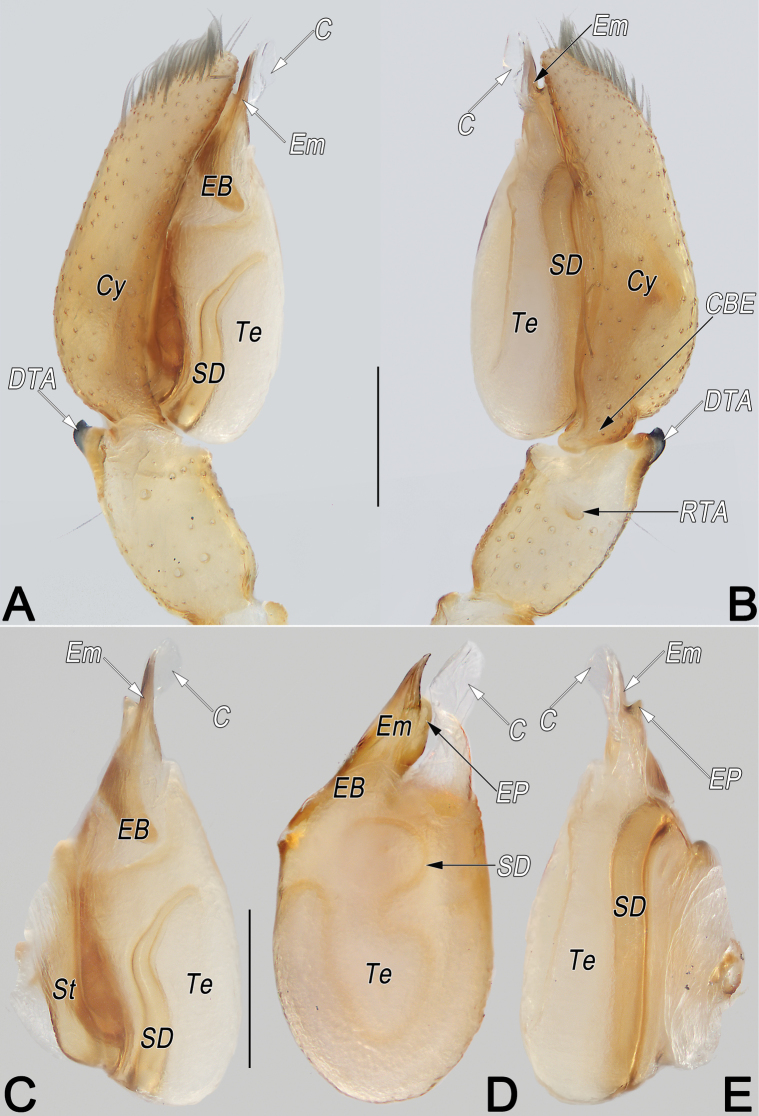
*Clubionahuntianling* sp. nov., holotype male palp. A. Prolateral view; B. Retrolateral view; C. Bulb, prolateral view; D. Bulb, ventral view; E. Bulb, retrolateral view. Abbreviations: C = conductor; CBE = cymbial base extension; Cy = cymbium; DTA = dorsal tibial apophysis; EB = embolic base; Em = embolus; EP = embolic projection; RTA = retrolateral tibial apophysis; SD = sperm duct; St = subtegulum; Te = tegulum; Scale bars: 0.2 mm.

**Figure 3.**
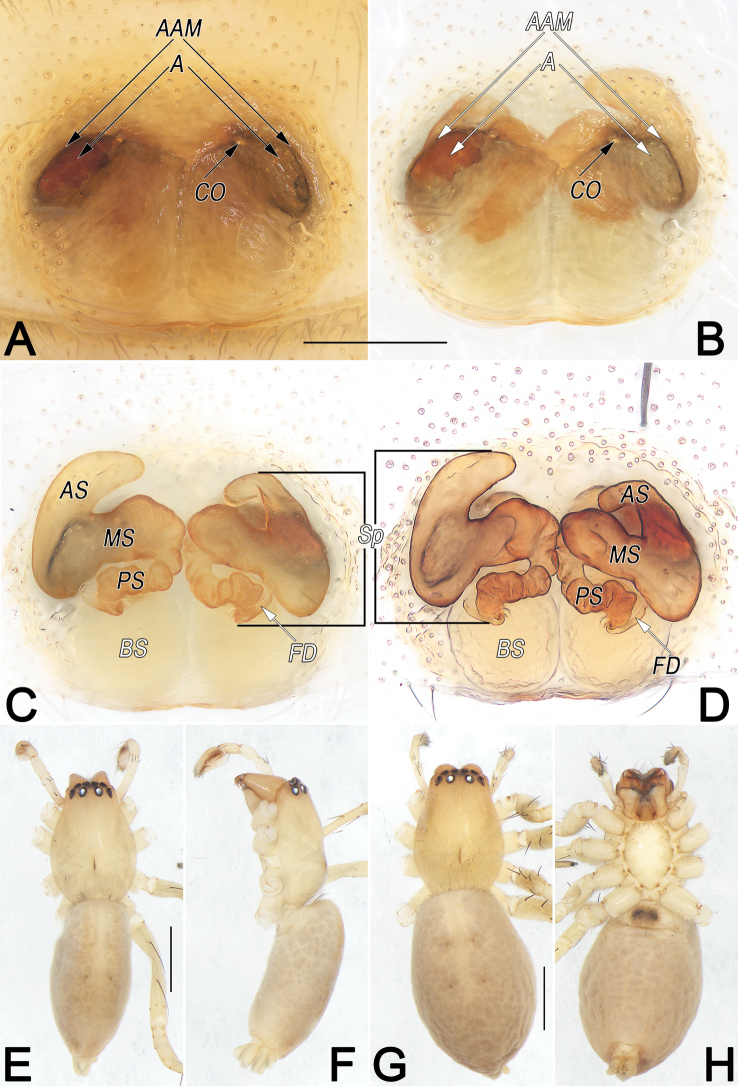
*Clubionahuntianling* sp. nov., female paratype and male holotype, epigyne (A–D), male habitus (E, F) and female habitus (G, H). A. Intact, ventral view; B. Cleared, ventral view; C. Cleared, dorsal view; D. Cleared, dorsal view; E. Dorsal view; F. Lateral view; G. Dorsal view; H. Ventral view. Abbreviations: A = atrium; AAM = atrial anterior margin; AS = anterior part of spermatheca; BS = bursa; CO = copulatory opening; FD = fertilisation duct; MS = median part of spermatheca; PS = posterior part of spermatheca; Sp = spermatheca. Scale bars: 0.2 mm (A–D); 1 mm (E–H).

Male of *Clubionahuntianling* sp. nov. resembles that of *C.pianmaensis* Wang, Wu & Zhang, 2015 in the general shape of male palp, but differs in the following: (1) sperm duct (SD) sinuous, resembling a long silk ribbon, narrowing and forming 3-shaped course in ventral view (vs not sinuous, wide and U-shaped) (cf. Fig. 2D and Wang et al. 2015: figs 7, 13); (2) tip of conductor (C) distinctly wider than embolic tip (vs nearly with same width) (cf. Fig. 2D and Wang et al. 2015: figs 7, 13). Female also resembles *C.pianmaensis* in having similarly shaped epigynes, but can be recognised by: (1) atrial anterior margin (AAM) less sclerotised, mesally faintly delimited (vs more sclerotised, entirely distinctly delimited) (cf. Fig. 3A, B and Wang et al. 2015: figs 3, 9); (2) median part of spermathecae (MS) unmodified (vs anterior surface with distinct hump) (cf. Fig. 3C, D and Wang et al. 2015: figs 4, 10).

##### Description.

**Male.** Holotype (Fig. 3E, F): Total length 4.53; carapace 1.93 long, 1.33 wide; abdomen 2.60 long, 1.23 wide. Carapace uniformly beige, without distinct pattern, fovea reddish; pars cephalica distinctly narrowed, cervical groove and radial grooves indistinct; tegument smooth, laterally and posteriorly with short, fine setae. AER slightly recurved, PER slightly wider than AER, almost straight in dorsal view. Eye sizes and interdistances: AME 0.10, ALE 0.13, PME 0.13, PLE 0.13, AME–AME 0.05, AME–ALE 0.03, PME–PME 0.16, PME–PLE 0.10, MOQL 0.31, MOQA 0.25, MOQP 0.39. Chelicerae pale orange, with three promarginal and two retromarginal teeth. Sternum off-white, 1.04 long, 0.78 wide. Labium and endites sandy coloured. Legs uniformly yellowish white, without distinct markings. Leg measurements: I 5.65 (1.45, 2.23, 1.23, 0.74), II 6.05 (1.70, 2.25, 1.39, 0.71), III 4.60 (1.32, 1.55, 1.25, 0.48), IV 6.48 (1.77, 2.17, 1.86, 0.68). Abdomen marked with numerous pale brown spots, dorsally with lengthwise beige heart mark reaching posterior half, with two pairs of brown muscle depressions laterally; venter medially with two longitudinal dotted lines.

Palp (Fig. 2A–E). Femur and patella unmodified. Tibia relatively long, ~1/2 of cymbium length, with two apophyses: dorsal apophysis (DTA) basally partly membranous, apically strongly sclerotised, pointing retrolatero-distally, ~1/5 of tibia length, more or less thumb-shaped; retrolateral apophysis (RTA) completely membranous, papilliform, distinctly small, ~1/2 of DTA length, pointing retrolatero-proximally. Cymbium (Cy) ~2.2× longer than wide, retrolaterally with basal extension (CBE), ~1/7–1/6 of cymbium length, consisting of inflated base and papilliform tip. Bulb elongated oval, slightly excavated on prolatero-apical side to accommodate embolus and conductor. Subtegulum (St) ~3/5 tegulum length, located dorso-prolaterally to tegulum. Tegulum (Te) oval, ~1.5× longer than wide; sperm duct (SD) sinuous, resembling a long silk ribbon, originating at retrolatero-distal flank (~1 o’clock position), aligning counter clockwise along tegulum margin, narrowing and forming 3-shaped course in ventral view. Embolus (Em) moderately sclerotised, ~2/3× of tegulum length and 1/4× of tegulum width, dagger-shaped; embolic base (EB) situated prolatero-distal flank of tegulum (~10–11 o’clock position); tip sharp, beak-shaped, slightly curved, pointing retrolatero-distally, terminating at ~12–1 o’clock position, subapically with flange-shaped projection (EP). Conductor (C) membranous, ~2/3 embolus length, extending alongside embolus, mesally strongly torqued along its length, tip wider than embolic tip.

**Female.** Paratype (Fig. 3G, H): Total length 5.10; carapace 1.99 long, 1.41 wide; abdomen 3.11 long, 1.97 wide. Eye sizes and interdistances: AME 0.11, ALE 0.13, PME 0.14, PLE 0.12, AME–AME 0.06, AME–ALE 0.04, PME–PME 0.18, PME–PLE 0.13, MOQL 0.30, MOQA 0.29, MOQP 0.45. Sternum 1.11 long, 0.82 wide. Leg measurements: I 4.20 (1.12, 1.71, 0.81, 0.56), II 4.43 (1.24, 1.79, 0.85, 0.55), III 3.78 (1.10, 1.31, 0.92, 0.45), IV 5.37 (1.45, 1.85, 1.50, 0.57). Slightly larger and darker than male, other characters as in male.

Epigyne (Fig. 3A–D). Epigynal plate slightly wider than long, margin not rebordered, spermathecae and bursae indistinctly visible through integument. Atrium (A) large, represented by two symmetrical, spherical, shallow depressions; atrial anterior margin (AAM) shaped like horizontally elongated lowercase m, mesally faintly delimited, lateral anterior margins rebordered; atrial posterior margin invisible. Copulatory openings (CO) small, located at atrial anterolateral borders, separated by ~1/3 of epigyne width. Spermathecae (Sp) with three parts: anterior part (AS) reniform, large, ~3× longer than wide; median part (MS) tubular, running horizontally; posterior part (PS) tubular, wrinkled, distinctly thinner than anterior and median parts of spermathecae. Bursae (BS) spherical, membranous, close together, ~1/2 of epigyne length, surface translucent and wrinkled. Fertilisation ducts (FD) short and curved, acicular, located distal end of spermathecal base.

##### Distribution.

Known only from the type locality, Mianning County, Sichuan, China (Fig. 1).

##### Etymology.

The specific name is derived from the Chinese pinyin ‘hùntiānlíng’, referring to a magical weapon of the youthful hero deity Nezha in ancient Chinese mythology, which is a long silk ribbon; a noun in apposition. This is in reference to the sinuate sperm duct of the new species, which is shaped like a long silk ribbon.

#### ﻿ Clubiona rouqiu

Taxon classificationAnimaliaAraneaeClubionidae

Yu & Li sp. nov.

D3B9ABB7-568D-5522-B393-0371BE308CA7

https://zoobank.org/2F1FD787-F09F-47CD-85C3-7A8A7E766ED9

Figs 1 , 4 , 5

##### Type material.

***Holotype***: China • ♂ (IZCAS-Ar 45531, YHCLU0393); Sichuan Prov., Panzhihua City, Miyi Co., Puwei Town, Ertaizi Vill.; 27.05°N, 101.99°E, ca 2266 m; 5.VI.2024; X. Zhang et al. leg. ***Paratype***: China • 1 ♀ (IZCAS-Ar 45532, YHCLU0382); Sichuan Prov., Liangshan Pref., Mianning Co., Yihai Town, Damawu Vill.; 28.61°N, 102.24°E, ca 2213 m; 9.VI.2024; X. Zhang et al. leg.

##### Diagnosis.

**Figure 4.**
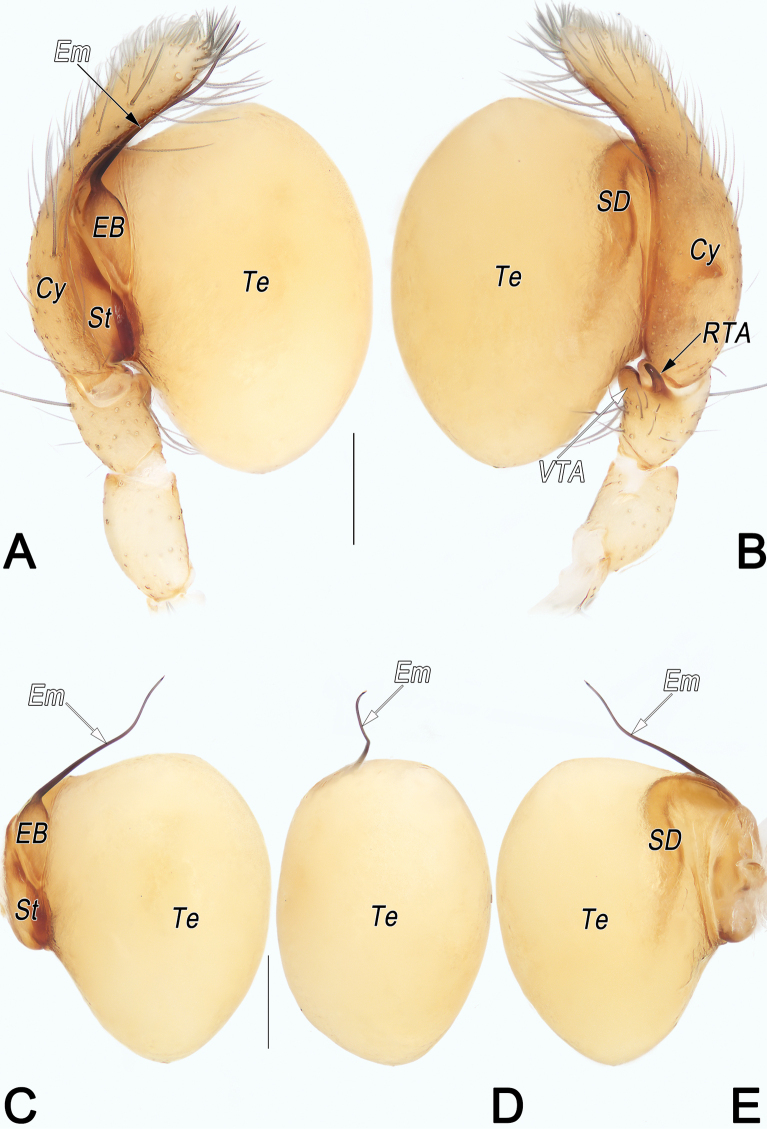
*Clubionarouqiu* sp. nov., holotype male palp. A. Prolateral view; B. Retrolateral view; C. Bulb, prolateral view; D. Bulb, ventral view; E. Bulb, retrolateral view. Abbreviations: Cy = cymbium; EB = embolic base; Em = embolus; RTA = retrolateral tibial apophysis; SD = sperm duct; St = subtegulum; Te = tegulum; VTA = ventral tibial apophysis. Scale bars: 0.2 mm.

**Figure 5.**
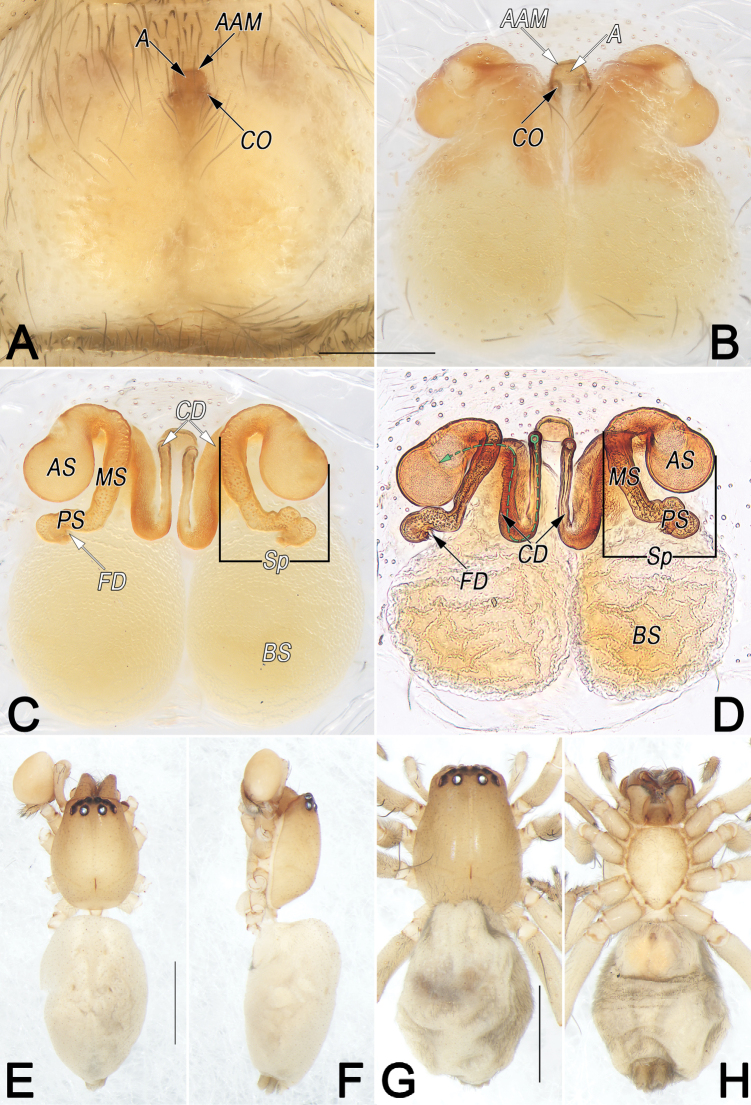
*Clubionarouqiu* sp. nov., female paratype and male holotype, epigyne (A–D), male habitus (E, F) and female habitus (G, H). A. Intact, ventral view; B. Cleared, ventral view; C. Cleared, dorsal view; D. Cleared, dorsal view (dashed line in D showing schematic course of copulatory duct, dorsal); E. Dorsal view; F. Lateral view; G. Dorsal view; H. Ventral view. Abbreviations: A = atrium; AAM = atrial anterior margin; AS = anterior part of spermatheca; BS = bursa; CD = copulatory duct; CO = copulatory opening; FD = fertilisation duct; MS = median part of spermatheca; PS = posterior part of spermatheca; Sp = spermatheca. Scale bars: 0.2 mm (A–D); 1 mm (E–H).

Male of *C.rouqiu* sp. nov. resembles that of *C.stiligera* Deeleman-Reinhold, 2001 (Deeleman-Reinhold 2001: figs 41, 42) in having similar filiform embolus, and distinctly inflated bulb, but differs by ventral tibial apophysis (VTA) papilliform (Fig. 4B) (vs subtriangular; Deeleman-Reinhold 2001: fig. 41); RTA thinner than VTA, slightly curved, tip pointing ventro-distally (Fig. 4B) (vs thicker, distinctly curved, tip pointing ventro-proximally; Deeleman-Reinhold 2001: fig. 41); embolus (Em) longitudinally extending, more or less ʃ-shaped in ventral view (Fig. 4D) (vs mainly horizontally extended, nearly ~-shaped; Deeleman-Reinhold 2001: fig. 42). Female of *C.rouqiu* sp. nov. resembles that of *C.tiane* Yu & Li, 2019, in having nearly arch-shaped atrium (A) and similarly shaped spermathecae (Sp) with tubular median and posterior parts (MS and PS), but can be recognised by globular anterior part (AS) (vs tubular), and by long and distinct copulatory ducts (CD) running along U-shaped course (vs short, indistinct, almost invisible) (cf. Fig. 5C, D and Zhang et al. 2021: fig. 19D, E).

##### Description.

**Male.** Holotype (Fig. 5E, F): Total length 3.73; carapace 1.43 long, 1.05 wide; abdomen 2.30 long, 1.27 wide. Carapace pale brown, slightly darker in cephalic area, with pair of short faint lines running longitudinally from behind AME, ocular region moderately narrowed; cervical groove indistinct; tegument smooth, clothed with short setae. AER slightly recurved, PER almost straight, latter wider than former. Eye sizes and interdistances: AME 0.08, ALE 0.10, PME 0.11, PLE 0.09, AME–AME 0.05, AME–ALE 0.02, PME–PME 0.13, PME–PLE 0.09, MOQL 0.24, MOQA 0.22, MOQP 0.33. Chelicerae coloured as ocular region, with three teeth on promargin and two teeth on retromargin. Sternum yellowish white, 0.80 long, 0.62 wide. Labium and endites pale orange. Legs uniformly yellowish white, without distinct markings. Leg measurements: I 2.79 (0.80, 1.14, 0.48, 0.37), II 3.21 (0.89, 1.31, 0.60, 0.41), III 2.77 (0.78, 0.92, 0.72, 0.35), IV 3.89 (1.12, 1.36, 1.01, 0.40). Abdomen oval, uniformly pale white, dorsally with lengthwise, double-edged spear-shaped beige heart mark, reaching posterior half, with two pairs of brown muscle depressions laterally; venter uniformly pale white, without distinct markings.

Palp (Fig. 4A–E). Femur and patella unmodified. Tibia short, ~1/4 of cymbium length, with two apophyses: ventral apophysis (VTA) slightly thicker, papilliform, ~1/4 of tibia length; retrolateral apophysis (RTA) relatively narrower, finger-shaped. Cymbium (Cy) distinctly slender, ~2.5× longer than wide. Bulb egg-shaped, distinctly inflated, strongly bulging and prolapsed, expanded posteriorly, reaching palpal patella. Subtegulum (St) ~2/5 of tegulum length, located dorso-prolaterally to tegulum. Tegulum (Te) ~1.45× longer than wide; sperm duct (SD) invisible in ventral view. Embolic base (EB) represented by enlarged tubercle, ~3/10 of tegulum length, situated meso-prolaterally on tegulum; free part of embolus (Em) slender, filiform, ~2/3 of tegulum length, tip slightly curved, sharp, extending above cymbium apex. Conductor absent.

**Female.** Paratype (Fig. 5G, H): Total length 3.45; carapace 1.55 long, 1.11 wide; abdomen 1.90 long, 1.39 wide. Eye sizes and interdistances: AME 0.09, ALE 0.11, PME 0.10, PLE 0.10, AME–AME 0.08, AME–ALE 0.02, PME–PME 0.18, PME–PLE 0.10, MOQL 0.26, MOQA 0.24, MOQP 0.37. Sternum 0.88 long, 0.65 wide. Leg measurements: I 2.60 (0.78, 1.00, 0.50, 0.32), II 2.77 (0.82, 1.07, 0.53, 0.35), III 2.59 (0.78, 0.86, 0.63, 0.32), IV 3.90 (1.16, 1.31, 1.07, 0.36). General characters as in male, but slightly smaller and darker.

Epigyne (Fig. 5A–D). Epigynal plate nearly as wide as long, margin not rebordered. Atrium (A) small, nearly trapezoidal, ~1/10 of epigyne width, atrial anterior margin (AAM) heavily sclerotised, posterior margin not delimited. Copulatory openings (CO) small, located at basolateral atrial borders. Copulatory ducts (CD) relatively long, descending longitudinally to vulva’s horizontal axis, then retracing, ascending longitudinally to form U-shaped course, finally extending laterally to connect with spermathecae. Spermathecae (Sp) with globular anterior part (AS), tubular median and posterior parts (MS and PS), small fertilisation ducts (FD) terminally; anterior part moderately large, ~1/5 of epigyne width, separated by 3× diameters; median part moderately long, ~1.5× longer than anterior part; posterior part wrinkled. Bursae (BS) close, nearly egg-shaped, ~2/3 of epigyne length, surface translucent, wrinkled.

##### Distribution.

Known only from the type localities, Miyi County and Mianning County, Sichuan, China (Fig. 1).

##### Etymology.

The specific name is derived from the Chinese pinyin ‘ròuqiú’, which means ‘flesh ball’, referring to the inflated, flesh-coloured palpal bulb of the new species, which is shaped like a flesh ball; noun in apposition. In Chinese traditional mythology, the youthful hero deity Nezha was born from a glowing flesh ball. The palpal bulb of the new species resembles this sphere.

#### ﻿ Clubiona yinyangjian

Taxon classificationAnimaliaAraneaeClubionidae

Yu & Li sp. nov.

BD5ADABC-BA65-5886-9280-49A4DED4AF77

https://zoobank.org/48CED5DF-726F-4A28-8AC7-BB3A027D43C7

Figs 1 , 6 , 7

##### Type material.

***Holotype***: China • ♂ (IZCAS-Ar 45533, YHCLU0395); Sichuan Prov., Panzhihua City, Miyi Co., Puwei Town, Ertaizi Vill.; 27.05°N, 101.99°E, ca 2266 m; 5.VI.2024; X. Zhang et al. leg. ***Paratype***: China • 1 ♀ (IZCAS-Ar 45534, YHCLU0396); Sichuan Prov., Panzhihua City, Miyi Co., Puwei Town, Pengjiayakou Vill.; 27.06°N, 102.00°E, ca 2464 m; 5.VI.2024; X. Zhang et al. leg.

##### Diagnosis.

**Figure 6.**
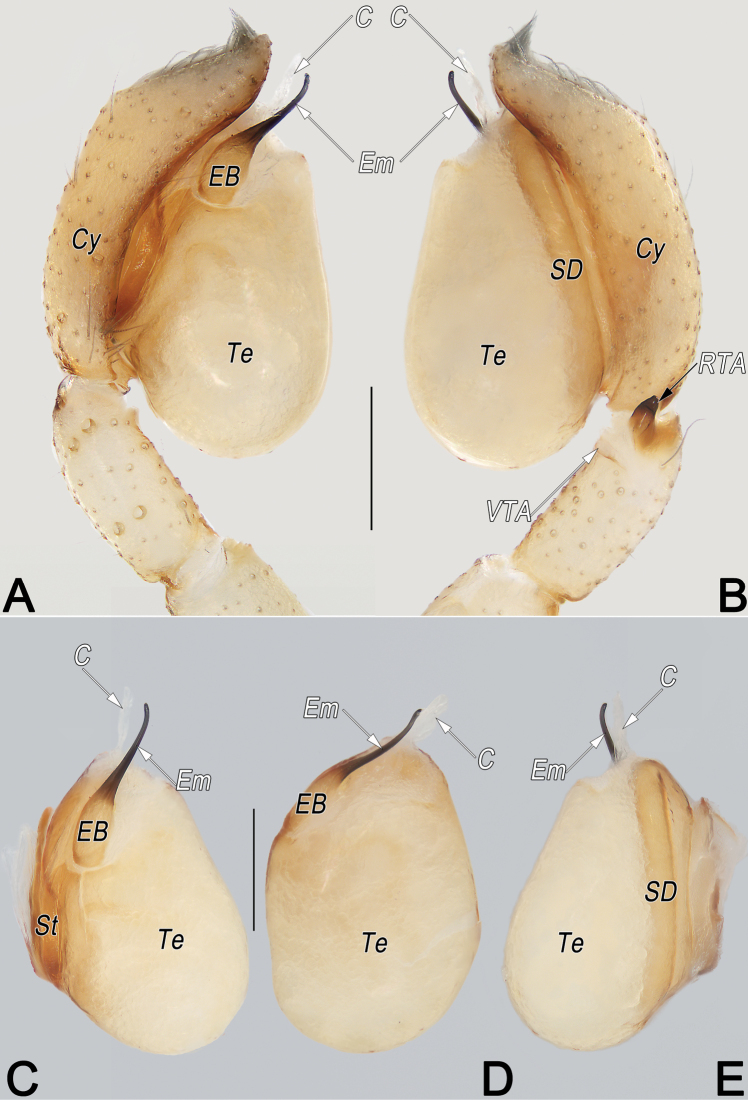
*Clubionayinyangjian* sp. nov., holotype male palp. A. Prolateral view; B. Retrolateral view; C. Bulb, prolateral view; D. Bulb, ventral view; E. Bulb, retrolateral view. Abbreviations: C = conductor; Cy = cymbium; EB = embolic base; Em = embolus; RTA = retrolateral tibial apophysis; SD = sperm duct; St = subtegulum; Te = tegulum; VTA = ventral tibial apophysis. Scale bars: 0.2 mm.

**Figure 7.**
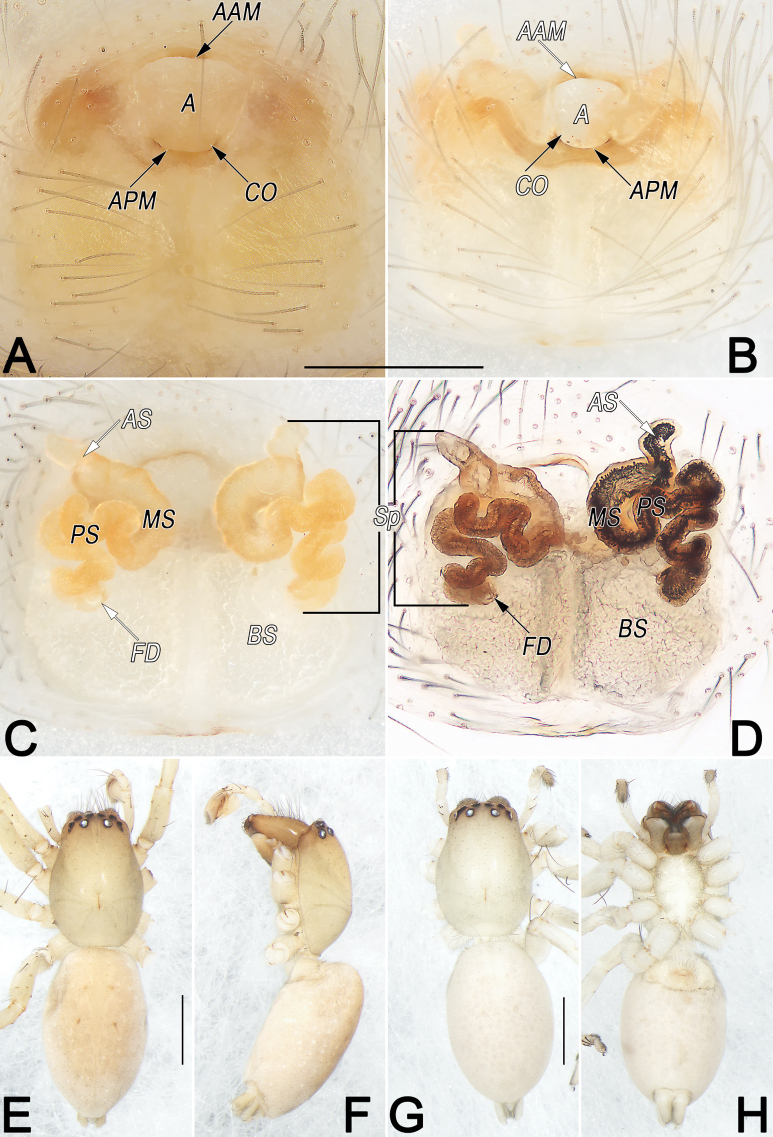
*Clubionayinyangjian* sp. nov., female paratype and male holotype, epigyne (A–D), male habitus (E, F) and female habitus (G, H). A. Intact, ventral view; B. Cleared, ventral view; C. Cleared, dorsal view; D. Cleared, dorsal view; E. Dorsal view; F. Lateral view; G. Dorsal view; H. Ventral view. Abbreviations: A = atrium; AAM = atrial anterior margin; APM = atrial posterior margin; AS = anterior part of spermatheca; BS = bursa; CO = copulatory opening; FD = fertilisation duct; MS = median part of spermatheca; PS = posterior part of spermatheca; Sp = spermatheca. Scale bars: 0.2 mm (A–D); 1 mm (E–H).

Male of the new species is easily distinguished from congroupers, with the exception of *C.tengchong* Zhang, Zhu & Song, 2007 (Zhang et al. 2007: fig. 2A–D), but can be separated from *C.tengchong* by embolus (Em) obliquely directed, tip pointing retrolateral-distally, terminating at ~1 o’clock position in ventral view (Em longitudinally erect, tip pointing distally, terminating at ~12 o’clock position) (cf. Fig. 6D and Zhang et al. 2007: fig. 2B); tegulum (Te) ~1.45× longer than wide (vs ~2×) (cf. Fig. 6D and Zhang et al. 2007: fig. 2B); sperm duct (SD) indistinct (vs distinct) (cf. Fig. 6D and Zhang et al. 2007: fig. 2B); ventral tibial apophysis (VTA) papilliform, apex blunt, partly membranous (vs digitiform, apex pointed, highly sclerotised) (cf. Fig. 6B and Zhang et al. 2007: fig. 2B–D). Female of *C.yinyangjian* sp. nov. is most similar to that of *C.yejiei* Yu & Li, 2021 by having similarly shaped atrium (A) and tubular spermathecae (Sp), but can be easily distinguished by: (1) anterior part of spermathecae (AS) horn-shaped, distinctly elevated, beyond atrial anterior margin (AAM) (vs claviform, slightly procurved, not overpassing AAM) (cf. Fig. 7C, D and Zhang et al. 2001: figs 23D, E, 89C); (2) bursae (BS) rounded square-shaped, nearly as long as wide (vs oblong, ~1.5× longer than wide) (cf. Fig. 7C, D and Zhang et al. 2001: figs 23D, E, 89C).

##### Description.

**Male.** Holotype (Fig. 7E, F): Total length 4.44; carapace 1.93 long, 1.37 wide; abdomen 2.51 long, 1.55 wide. Carapace more or less pyriform, pale grey except pale brown ocular area, without pattern; ocular area slightly narrowed, in profile highest just behind longitudinal fovea, cervical groove distinct, radial grooves indistinct; tegument smooth, laterally and posteriorly clothed with short, fine setae. Eyes: both AER and PER slightly recurved in dorsal view, latter wider than former. Eye sizes and interdistances: AME 0.14, ALE 0.12, PME 0.13, PLE 0.14, AME–AME 0.06, AME–ALE 0.04, PME–PME 0.23, PME–PLE 0.15, MOQL 0.33, MOQA 0.31, MOQP 0.48. Chelicerae robust, pale orange, with three promarginal and two retromarginal teeth. Sternum off-white, 1.04 long, 0.77 wide. Labium uniformly dark brown. Endites proximally pale brown, distally off-white. Legs uniformly yellowish white, without distinct markings. Leg measurements: I 5.00 (1.38, 2.04, 1.04, 0.54), II 5.64 (1.58, 2.28, 1.20, 0.58), III 4.33 (1.22, 1.53, 1.16, 0.42), IV 6.14 (1.67, 2.09, 1.76, 0.62). Abdomen oval, dorsally beige with wide, brownish scutum extending ~1/5 of abdomen length, with two pairs of brown muscle depressions located at the central part of scutum; venter medially with two indistinct longitudinal dotted linear markings.

Palp (Fig. 6A–E). Femur and patella unmodified. Tibia relatively long, ~1/2 of cymbium length, with two apophyses: retrolateral apophysis (RTA) heavily sclerotised, ~1/4 of palpal tibia length, with narrowed tip, thumb-like; ventro-retrolateral apophysis (VTA) small, papilliform, partly membranous. Cymbium (Cy) ~2.1× longer than wide, unmodified. Bulb nearly pyriform, proximally distinctly bulging, prolapsed, proapically and apically membranous, slightly excavated to accommodate embolus and conductor. Subtegulum (St) ~1/2 tegulum length, located dorso-prolaterally to tegulum. Tegulum (Te) elongate, oval, ~1.45× longer than wide; sperm duct (SD) indistinct in ventral and prolateral views. Embolus (Em) sword-shaped, relatively long, ~3/5 of tegulum length; embolic base (EB) represented by enlarged tubercle, originating at prolatero-distal portion of tegulum (~11 o’clock), gradually tapering toward tip; tip slightly curved, pointing retrolateral-distally, terminating at ~1 o’clock position. Conductor (C) nearly as long as embolus, white and membranous, extending alongside embolus.

**Female.** Paratype (Fig. 7G, H): Total length 4.46; carapace 2.03 long, 1.47 wide; abdomen 2.43 long, 1.60 wide. Eye sizes and interdistances: AME 0.12, ALE 0.14, PME 0.13, PLE 0.13, AME–AME 0.08, AME–ALE 0.07, PME–PME 0.25, PME–PLE 0.14, MOQL 0.30, MOQA 0.33, MOQP 0.50. Sternum 1.10 long, 0.77 wide. Leg measurements: I 3.97 (1.09, 1.66, 0.72, 0.50), II 4.31 (1.25, 1.74, 0.81, 0.51), III 3.66 (1.10, 1.33, 0.91, 0.32), IV 5.31 (1.42, 1.88, 1.53, 0.48). Similar to male but general colouration distinctly paler than in male.

Epigyne (Fig. 7A–D). Epigynal plate nearly as wide as long. Atrium (A) ~3/10 of epigyne length and width, more or less apple-shaped, both anterior margin (AAM) and posterior margin (APM) distinctly sclerotised. Copulatory openings (CO) indistinct, located at basolateral atrial borders. Copulatory ducts (CD) indistinct, entirely covered by large, tubular spermathecae (Sp). Spermathecae long and sinuous, consisting of horn-shaped anterior part (AS), arc-shaped median part (MS) and convoluted posterior part (PS), with small fertilisation ducts (FD) terminally; anterior and median parts of spermathecae rise and curl up to form two folds, median parts separated by one diameter. Bursae (BS) rounded square-shaped, relatively large, ~1/2 of epigyne length, close together, surface translucent, wrinkled.

##### Distribution.

Known only from the type locality, Miyi County, Sichuan, China (Fig. 1).

##### Etymology.

The specific name is derived from the Chinese pinyin ‘yīnyángjiàn’, referring to a pair of swords of the youthful hero deity Nezha in ancient Chinese mythology, consisting of one black sword and one white sword; a noun in apposition. This name is in reference to the black, strongly sclerotised embolus and the white, membranous conductor of the new species, both of which are elongated and sword-shaped.

### ﻿*Clubionatrivialis* group

**Diagnosis.***Clubionatrivialis* group and *C.zilla* group share the combination of following characters of copulatory organs (such combination of structures of copulatory organs has never been observed in any other species groups): retrolateral tibial apophysis simple, erect, and lacking prominent dentition; tegulum ventrally with distinct, meandering sperm duct, retrolaterally with partly membranous groove; embolic base typically bears 1–3 tooth-like processes; free part of embolus flagelliform, arched around or angled across the distal end of tegulum; conductor reduced; epigynal plate with strongly sclerotised posterior margin extending beyond epigastric furrow; copulatory openings positioned posteriorly, joined medially or spaced closely; copulatory ducts slender, running parallel and juxtaposed at midline, then extending laterally in straight or arched paths. Members of the *trivialis* group can be separated from those of the *zilla* group by: (1) with larger bodies (length at least 5 mm) (vs with tiny bodies < 3.6 mm); (2) embolic base processes small, distinctly shorter than embolic base (vs developed, nearly as long as embolic base); (3) epigyne comprises large ventral plate and semi-transparent dorsal plate, both primary and secondary spermathecae sandwiched between dorsal and ventral plates (vs dorsal plate absent); (4) epigyne without hood (or guide pockets) (vs presence of hood (or guide pockets) near the copulatory openings).

**Comments.***Microclubiona* Lohmander, 1944 was described to accommodate species related to *Clubionatrivialis* C. L. Koch, 1843 (type species). However, his work was largely overlooked. *Clubionatrivialis* and its allied species were subsequently assigned to Group III in Locket and Millidge (1951) and to Group I in Edwards (1958). The *trivialis* group was formally named by Dondale and Redner (1976) and later revised by Mikhailov (1995), who defined it based on 19 Holarctic species. The group was resurrected to the genus rank by Wunderlich (2011) but was later synonymized with *Clubiona* by Mikhailov (2012). The present study follows Mikhailov (2012) and WSC (2025) in treating *Microclubiona* as a synonym of *Clubiona*, rather than reinstating it as a separate genus.

To date, the most comprehensive global checklist of the *trivialis* group has been provided by Zhang et al. (2021). Since then, two additional species have been described as new: *C.flammaformis* L.F. Li, Liu, B. Li & Peng, 2023 and *C.bi* Zhang, Zhong & Gong, 2024, both endemic to China (Li et al. 2023; Zhang et al. 2024).

#### ﻿ Clubiona huojianqiang

Taxon classificationAnimaliaAraneaeClubionidae

Yu & Li sp. nov.

DC08B945-4923-5B0D-8B0F-872842AB4F9C

https://zoobank.org/B60928A4-48DB-4581-9A7F-897C52F55B77

Figs 1 , 8 , 9

##### Type material.

***Holotype***: China • ♂ (IZCAS-Ar 45535); Sichuan Prov., Liangshan Pref., Mianning Co., Senrong Town, Wulibei Vill.; 28.37°N, 101.99°E, ca 2462 m; 10.VI.2024; X. Zhang et al. leg. ***Paratype***: China • 1 ♀ (IZCAS-Ar 45536); same data as for holotype.

##### Other material examined.

China • 1 ♀ (YHCLU0402); Sichuan Prov., Liangshan Pref., Mianning Co., Yihai Town, Damawu Vill.; 28.61°N, 102.24°E, ca 2213 m; 9.VI.2024; X. Zhang et al. leg.

##### Diagnosis.

**Figure 8.**
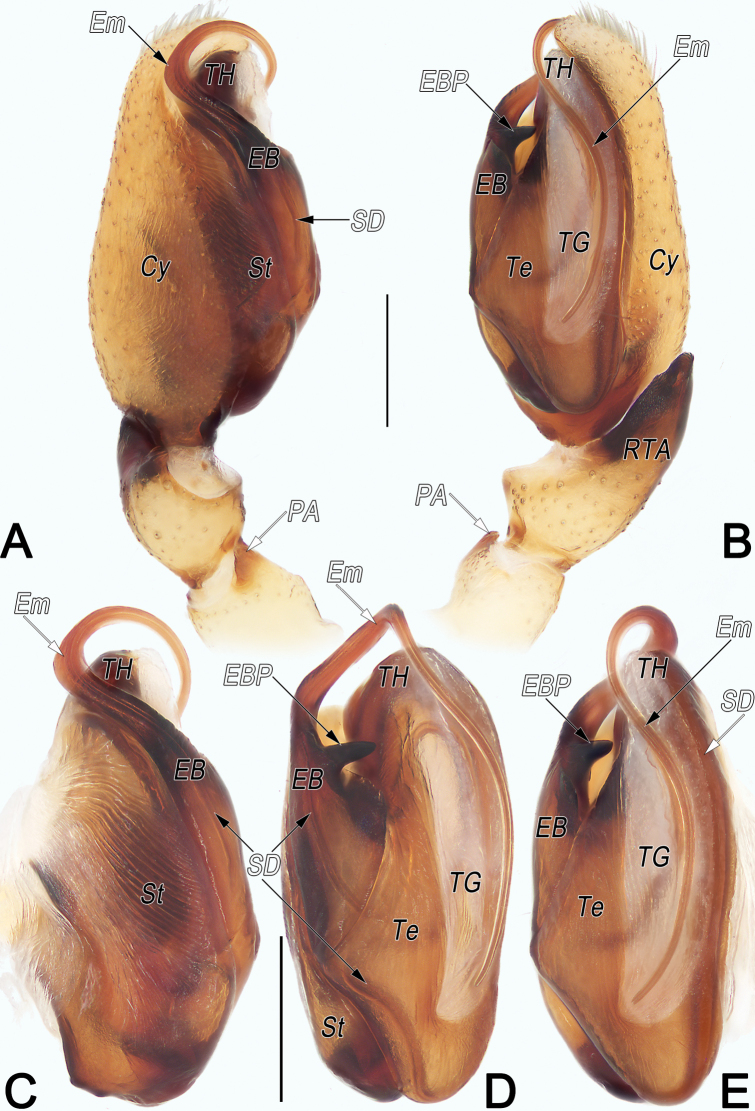
*Clubionahuojianqiang* sp. nov., holotype male palp. A. Prolateral view; B. Retrolateral view; C. Bulb, prolateral view; D. Bulb, ventral view; E. Bulb, retrolateral view. Abbreviations: Cy = cymbium; EB = embolic base; EBP = embolic base process; Em = embolus; PA = patellar apophysis; RTA = retrolateral tibial apophysis; SD = sperm duct; St = subtegulum; Te = tegulum; TG = tegular groove; TH = tegular hump. Scale bars: 0.2 mm.

**Figure 9.**
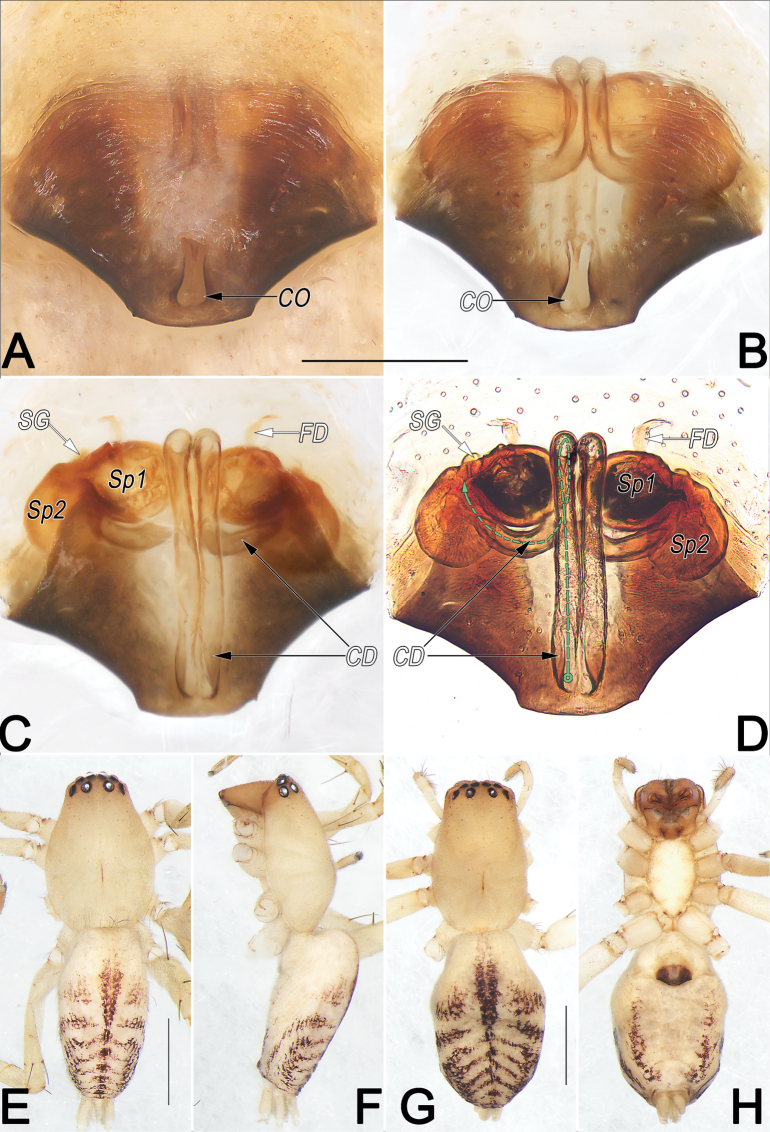
*Clubionahuojianqiang* sp. nov., female paratype and male holotype, epigyne (A–D), male habitus (E, F) and female habitus (G, H). A. Intact, ventral view; B. Cleared, ventral view; C. Cleared, dorsal view; D. Cleared, dorsal view (dashed line in D showing schematic course of copulatory duct, dorsal); E. Dorsal view; F. Lateral view; G. Dorsal view; H. Ventral view. Abbreviations: CD = copulatory duct; CO = copulatory opening; FD = fertilisation duct; SG = spermathecal gland; Sp1 = primary spermatheca; Sp2 = secondary spermatheca. Scale bars: 0.2 mm (A–D); 1 mm (E–H).

Both sexes of *C.huojianqiang* sp. nov. are very similar to those of *C.contrita* Forster, 1979 in having similar habitus, palps and epigynes (see Figs 8, 9 and Forster 1979: figs 289, 297–298, 304–307 and Paquin et al. 2010: fig. 41.2–3). From *C.contrita*, the male can be distinguished by: embolic base process (EBP) protruding horizontally, apex pointing retrolaterally (vs protruding longitudinally, apex pointing distally) (cf. Fig. 8B, D, E and Forster 1979: fig. 304 and Paquin et al. 2010: fig. 41.2), and retrolateral tibial apophysis (RTA) distinctly shorter, ~1.2–1.3× longer than tibia (vs distinctly longer, ~2× longer than tibia) (cf. Fig. 8B and Forster 1979: figs 304, 305 and Paquin et al. 2010: fig. 41.2–3); the female can be recognised by: both primary (Sp1) and secondary spermathecae (Sp2) globular (vs Sp1 diamond-shaped, Sp2 bean-shaped) (cf. Fig. 9C, D and Forster 1979: fig. 307).

##### Description.

**Male.** Holotype (Fig. 9E, F): Total length 3.74; carapace 1.81 long, 1.25 wide; abdomen 1.93 long, 1.03 wide. Carapace uniformly yellowish white, without distinct pattern, fovea reddish; cephalic region distinctly narrowed, slightly darker, cervical groove and radial grooves invisible; tegument smooth, marginally clothed with short, fine setae. Eyes: both AER and PER slightly recurved in dorsal view, latter wider than former. Eye sizes and interdistances: AME 0.10, ALE 0.11, PME 0.10, PLE 0.12, AME–AME 0.05, AME–ALE 0.03, PME–PME 0.18, PME–PLE 0.11, MOQL 0.30, MOQA 0.24, MOQP 0.38. Chelicerae robust, pale orange, with four promarginal and five retromarginal teeth. Sternum yellowish white, 0.98 long, 0.64 wide. Labium and endites pale orange. Legs coloured as carapace, without distinct markings. Leg measurements: I 3.98 (1.10, 1.60, 0.84, 0.44), II 4.08 (1.14, 1.64, 0.85, 0.45), III 3.55 (1.02, 1.28, 0.85, 0.40), IV 5.29 (1.54, 1.82, 1.41, 0.52). Abdomen elongate, oval, with thick tuft of setae anteriorly; dorsum medially with broken brown longitudinal band, starting anteriorly for half the length; seven or eight pairs of broken lateral bands fused posteriorly. Venter uniformly creamy white, without markings.

Palp (Fig. 8A–E). Patella with distinct, subtriangular, small ventral apophysis (PA) originating distally, ~1/3 of patella length. Tibia short, ~1/4 of cymbium (Cy) length; retrolateral tibial apophysis (RTA) ~1.2–1.3× tibia length, triangular in lateral views, both ventral and dorsal edges with countless delicate texture. Subtegulum (ST) large, nearly as long as tegulum, prolateral surface partly membranous, wrinkled and ribbed, with numerous diagonal ridges. Tegulum (Te) elongated-oval, ~2× longer than wide, distally with distinctly, semicircular hump (TH); sperm duct (SD) distinct and sinuous, its course like a horizontally placed capital letter S. Embolic base (EB) long and flat, located at ~8–10 o’clock position of tegulum, retrolateral-distally bearing tooth-shaped process (EBP); EBP ~1/5 of embolic base length, apex pointing retrolaterally; free part of embolus (Em) slender, flagelliform, at right angle ventrally and angled across tegular hump (TH), stretched proximally along membranous tegular groove, tip extending to 3/4 of tegulum length. Tegular groove (TG) relatively large, ~2/5 tegulum width and 4/5 tegulum length.

**Female.** Paratype (Fig. 9G, H): Total length 4.22; carapace 1.90 long, 1.20 wide; abdomen 2.32 long, 1.45 wide. Eye sizes and interdistances: AME 0.11, ALE 0.10, PME 0.10, PLE 0.10, AME–AME 0.07, AME–ALE 0.05, PME–PME 0.21, PME–PLE 0.14, MOQL 0.26, MOQA 0.27, MOQP 0.40. Sternum 1.03 long, 0.65 wide. Leg measurements: I 3.33 (0.90, 1.38, 0.64, 0.41), II 3.52 (1.01, 1.39, 0.70, 0.42), III 3.26 (0.97, 1.15, 0.78, 0.36), IV 4.93 (1.43, 1.68, 1.31, 0.51). Slightly larger and darker than male, other characters as in male.

Epigyne (Fig. 9A–D). Epigynal plate ~1.5× wider than long, anterior and lateral margins not rebordered; posterior margin heavily sclerotised, convex medially, with posteriorly protruding edge, forming inverted trapezoidal outline; spermathecae and copulatory ducts prominently visible through tegument in ventral view. Copulatory openings (CO) large, conjoined, located at posterior portion of epigynal plate, keyhole-shaped, distinctly narrow, ~1/5 of epigyne length and 1/13 of epigyne width. Copulatory ducts (CD) long, slender, almost parallel and ascending dorsally, extending above anterior surface of primary spermathecae (Sp1), then retracing ventrally, descending vertically under posterior surface of primary spermathecae, finally curving laterally to connect with secondary spermathecae (Sp2). Both primary and secondary spermathecae globular and of similar size, with former located anteromedially to latter. Primary spermathecae close together, moderately large, diameter ~1/5 of epigyne width, inside pigmented and sclerotised, surface hyaline; spermathecal gland (SG) distinctly small, papilliform, located at anterolateral surfaces of primary spermathecae. Secondary spermathecae surface translucent, smooth, separated by ~1.9 diameters. Fertilisation ducts (FD) acicular, ~1/2 of diameter of primary spermathecae, on their ventral surfaces.

##### Distribution.

Known only from the type locality, Mianning County, Sichuan, China (Fig. 1).

##### Etymology.

The specific name is derived from the Chinese pinyin ‘huǒjiānqiāng’, referring to a magical weapon of the youthful hero deity Nezha in ancient Chinese mythology, which is a sharp spear; a noun in apposition. This name refers to the embolic base process of the new species, which resembles a sharp spearhead.

#### ﻿ Clubiona qiankunquan

Taxon classificationAnimaliaAraneaeClubionidae

Yu & Li sp. nov.

2E75AAB4-6D2C-5981-B53A-CA0C7433DD34

https://zoobank.org/63080518-97A6-4F73-826F-4A6F24552410

Figs 1 , 10 , 11

##### Type material.

***Holotype***: China • ♂ (IZCAS-Ar 45537, YHCLU0385); Sichuan Prov., Panzhihua City, Miyi Co., Puwei Town, Pengjiayakou Vill.; 27.06°N, 102.00°E, ca 2464 m; 5.VI.2024; X. Zhang et al. leg. ***Paratype***: China • 1 ♀ (IZCAS-Ar 45538, YHCLU0386); same data as for holotype.

##### Diagnosis.

**Figure 10.**
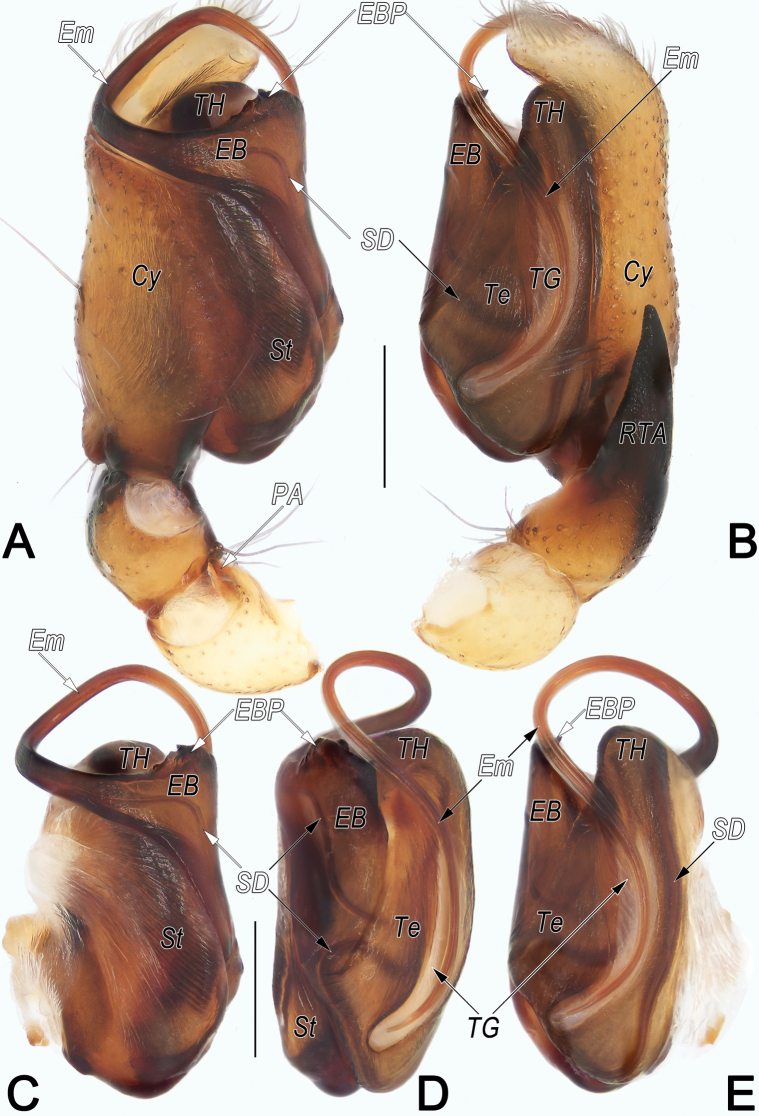
*Clubionaqiankunquan* sp. nov., holotype male palp. A. Prolateral view; B. Retrolateral view; C. Bulb, prolateral view; D. Bulb, ventral view; E. Bulb, retrolateral view. Abbreviations: Cy = cymbium; EB = embolic base; EBP = embolic base process; Em = embolus; PA = patellar apophysis; RTA = retrolateral tibial apophysis; SD = sperm duct; St = subtegulum; Te = tegulum; TG = tegular groove; TH = tegular hump. Scale bars: 0.2 mm.

**Figure 11.**
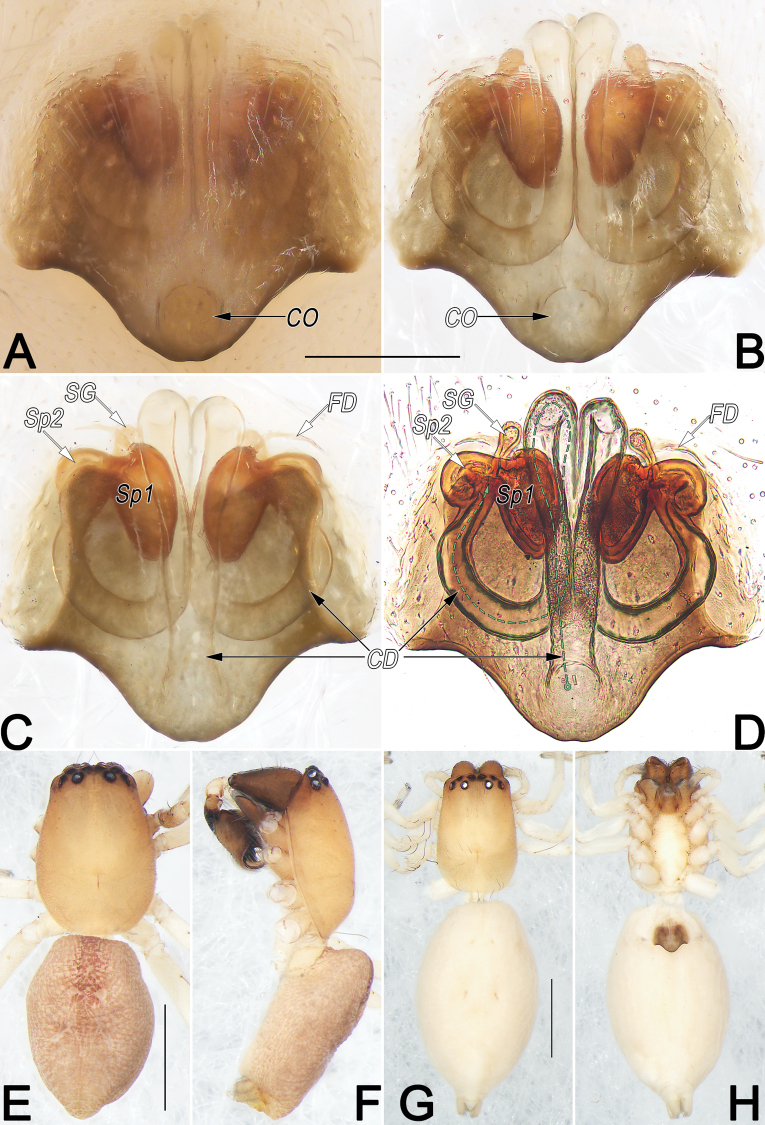
*Clubionaqiankunquan* sp. nov., female paratype and male holotype, epigyne (A–D), male habitus (E, F) and female habitus (G, H). A. Intact, ventral view; B. Cleared, ventral view; C. Cleared, dorsal view; D. Cleared, dorsal view (dashed line in D showing schematic course of copulatory duct, dorsal); E. Dorsal view; F. Lateral view; G. Dorsal view; H. Ventral view. Abbreviations: CD = copulatory duct; CO = copulatory opening; FD = fertilisation duct; SG = spermathecal gland; Sp1 = primary spermatheca; Sp2 = secondary spermatheca. Scale bars: 0.2 mm (A–D); 1 mm (E–H).

Male of *C.qiankunquan* sp. nov. resembles that of *C.subasrevida* Yu & Li, 2019 in the shape of male palp, but differs in following: (1) loop-like torsion of embolus (Em) in ventral view distinctly larger, diameter ~1/2 of tegulum width (vs smaller, ≤ 1/4 of tegulum width) (cf. Fig. 10D and Yu and Li 2019b: fig. 17D); (2) embolus longer, tip extending basad > 4/5 of tegulum length (vs ~2/3 tegulum length) (cf. Fig. 10B, D, E and Yu and Li 2019b: fig. 17B, D, E). Female also resembles those of *C.subasrevida*, but can be distinguished by the following: (1) posterior margin of epigynal plate distinctly protruded, with length of protrusion ~1/3 of epigyne length (vs slightly protruded, with protrusion < 1/7 of epigyne length) (cf. Fig. 11A–D and Yu and Li 2019b: fig. 18A–D); (2) primary spermathecae (Sp1) oval, located medially to secondary spermathecae (Sp2) (vs Sp1 globular, located anteriorly to Sp2) (cf. Fig. 11C, D and Yu and Li 2019b: fig. 18C, D); (3) spermathecal gland (SG) clubbed (vs horn-shaped) (cf. Fig. 11C, D and Yu and Li 2019b: fig. 18C, D).

##### Description.

**Male.** Holotype (Fig. 11E, F): Total length 3.45; carapace 1.64 long, 1.16 wide; abdomen 1.81 long, 1.23 wide. Carapace pale orange, slightly darker front ridge, without pattern, fovea reddish; ocular area slightly narrowed, cervical groove and radial grooves indistinct; tegument smooth, laterally and posteriorly clothed with short, fine setae. Eyes: in dorsal view, AER slightly recurved, PER almost straight, PER slightly wider than AER. Eye sizes and interdistances: AME 0.11, ALE 0.12, PME 0.11, PLE 0.11, AME–AME 0.05, AME–ALE 0.06, PME–PME 0.23, PME–PLE 0.12, MOQL 0.26, MOQA 0.26, MOQP 0.42. Chelicerae robust, dark brown, promargin and retromargin with three teeth. Sternum slightly paler than carapace, 0.90 long, 0.63 wide. Labium and endites dark brown. Legs uniformly pale white, without distinct markings. Leg measurements: I 2.79 (0.79, 1.17, 0.58, 0.25), II 2.98 (0.85, 1.25, 0.57, 0.31), III 2.52 (0.74, 0.88, 0.62, 0.28), IV 3.52 (1.06, 1.26, 0.85, 0.35). Abdomen oval, dorsally pale brown with reddish brown, lanceolate-shaped median band, reaching posterior half; venter uniformly yellowish white.

Palp (Fig. 10A–E). Patella distally with small, nearly cone-shaped ventro-prolateral apophysis (PA) that ~1/3 of patella length. Tibia short, ~1/4 of cymbium (Cy) length; retrolateral tibial apophysis (RTA) ~2× longer than tibia, shovel-shaped in ventral view, blade-shaped in retrolateral view. Subtegulum (ST) large, ~4/5 tegulum length, prolateral surface hyaline, wrinkled, ribbed, with numerous diagonal ridges. Tegulum (Te) oblong in ventral view, ~1.9× longer than wide; tegular hump (TH) humble; sperm duct (SD) distinct, sinuous, running irregular course that meandering with at least four turns or loops before entering embolic base in ventral view. embolic base (EB) large, ~1/2 of tegulum width and 2/3 of tegulum length, distally elevated to form process (EBP); EBP small, represented by indistinct hump, with several ridges and a tooth; free part of embolus (Em) flagelliform, aligned transversely on apical part of bulb, forming loop-shaped torsion; tip slightly curved, stretched proximally on tegular groove, extending basad > 4/5 of tegulum length. Tegular groove (TG) relatively long, narrow, shaped like symbol) ~3/5 of tegulum length and 1/6 of tegulum width.

**Female.** Paratype (Fig. 11G, H): Total length 3.98; carapace 1.49 long, 1.00 wide; abdomen 2.49 long, 1.53 wide. Eye sizes and interdistances: AME 0.09, ALE 0.11, PME 0.10, PLE 0.09, AME–AME 0.06, AME–ALE 0.06, PME–PME 0.23, PME–PLE 0.11, MOQL 0.23, MOQA 0.25, MOQP 0.41. Sternum 0.91 long, 0.54 wide. Leg measurements: I 2.45 (0.71, 1.00, 0.44, 0.30), II 2.70 (0.82, 1.08, 0.53, 0.27), III 2.33 (0.71, 0.80, 0.56, 0.26), IV 3.47 (1.01, 1.26, 0.85, 0.35). Similar to male but distinctly larger and paler.

Epigyne (Fig. 11A–D). Epigynal plate nearly as long as wide, anterior and lateral margins not delimited, posterior margin rebordered, heavily sclerotised and convex, medially with downward-protruding edge, forming nearly inverted triangular outline; arrangement of various parts of endogyne indistinctly visible through tegument. Copulatory openings (CO) circular, large, ~1/6 of epigyne length, completely fused along axis, situated at protruding portion of posterior margin of epigynal plate. Copulatory ducts (CD) hyaline, ascending dorsally, proximally close together at midline, distally slightly convergent, extending to anterior margin of epigyne; then retracing ventrally, descending vertically, smoothly bending outward in curved arc, following J-shaped course; finally extending diagonally upward, entering connecting piece between primary and secondary spermathecae. Primary spermathecae (Sp1) oval, ~3/10 epigyne length, 1.7× longer than wide, moderately sclerotised, separated by < 1 radius; spermathecal gland (SG) clubbed, distally slightly inflated, located at anterolateral surface of primary spermathecae. Secondary spermathecae (Sp2) located laterally to primary spermathecae, globular, surface hyaline. Fertilisation ducts (FD) acicular, relatively long, nearly equal to length of primary spermathecae, located anteroventral surface of primary spermathecae.

##### Distribution.

Known only from the type locality, Miyi County, Sichuan, China (Fig. 1).

##### Etymology.

The specific name is derived from the Chinese pinyin ‘qiánkūnquān’, referring to a magical weapon of the youthful hero deity Nezha in ancient Chinese mythology, which is a circular metal ring; a noun in apposition. This name refers to the embolus of the new species, which forms a loop in ventral view.

### ﻿*Clubionazilla* group

**Diagnosis.** Members of the *zilla* group can be distinguished from those of all other species groups with the exception of the *trivialis* group. For similarities and differences between the two groups, see the diagnosis for the *trivialis* group above.

**Comments.***Clubionazilla* group was established by Ono (1986). Mikhailov (1995) later revised the definition of the group, and Ono (2010) elevated it to the genus *Anaclubiona* to accommodate three species: *A.zilla* Dönitz & Strand, 1906, *A.minima* Ono, 2010, and *A.tanikawai* (Ono, 1989). However, Mikhailov (2012) synonymised *Anaclubiona*. Recently, a new species, *C.jiugong* Yu & Zhong, 2021 was described in this group, and *C.hooda* Dong & Zhang, 2016 was assigned to this group by Zeng et al. (2021). In conclusion, the *zilla* group currently includes at least five species distributed exclusively in China and Japan.

#### ﻿ Clubiona nezha

Taxon classificationAnimaliaAraneaeClubionidae

Yu & Li sp. nov.

0C70DBE5-5864-580E-A6FE-BA11CCAAA4E4

https://zoobank.org/F8D597CC-DB3E-42D6-A7F8-C5BBF5BA1E09

Figs 1 , 12 , 13

##### Type material.

***Holotype***: China • ♂ (IZCAS-Ar 45539, YHCLU0391); Sichuan Prov., Mianyang City, Jiangyou County-level City, Yongsheng Town, Xinbei Vill.; 31.94°N, 104.81°E, ca 681 m; 16.V.2024; X. Zhang et al. leg. ***Paratype***: China • 1 ♀ (IZCAS-Ar 45540, YHCLU0392); same data as for holotype.

##### Diagnosis.

**Figure 12.**
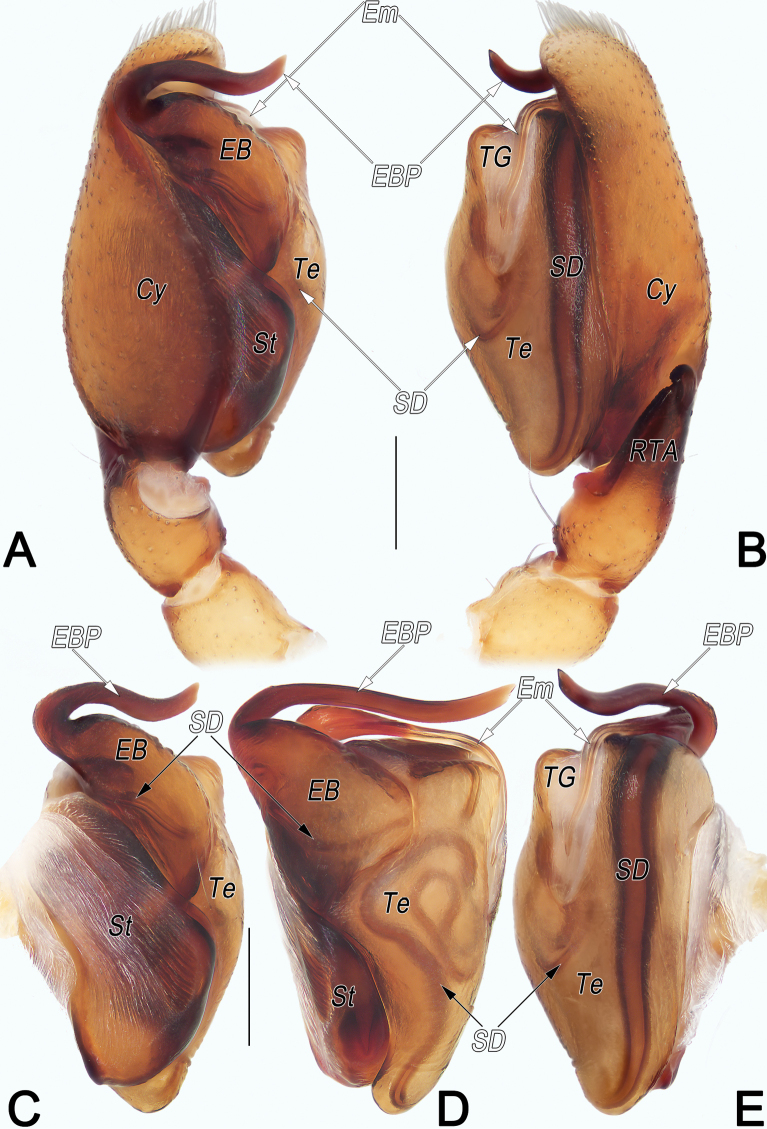
*Clubionanezha* sp. nov., holotype male palp. A. Prolateral view; B. Retrolateral view; C. Bulb, prolateral view; D. Bulb, ventral view; E. Bulb, retrolateral view. Abbreviations: Cy = cymbium; EB = embolic base; EBP = embolic base process; Em = embolus; RTA = retrolateral tibial apophysis; SD = sperm duct; St = subtegulum; Te = tegulum; TG = tegular groove. Scale bars: 0.2 mm.

**Figure 13.**
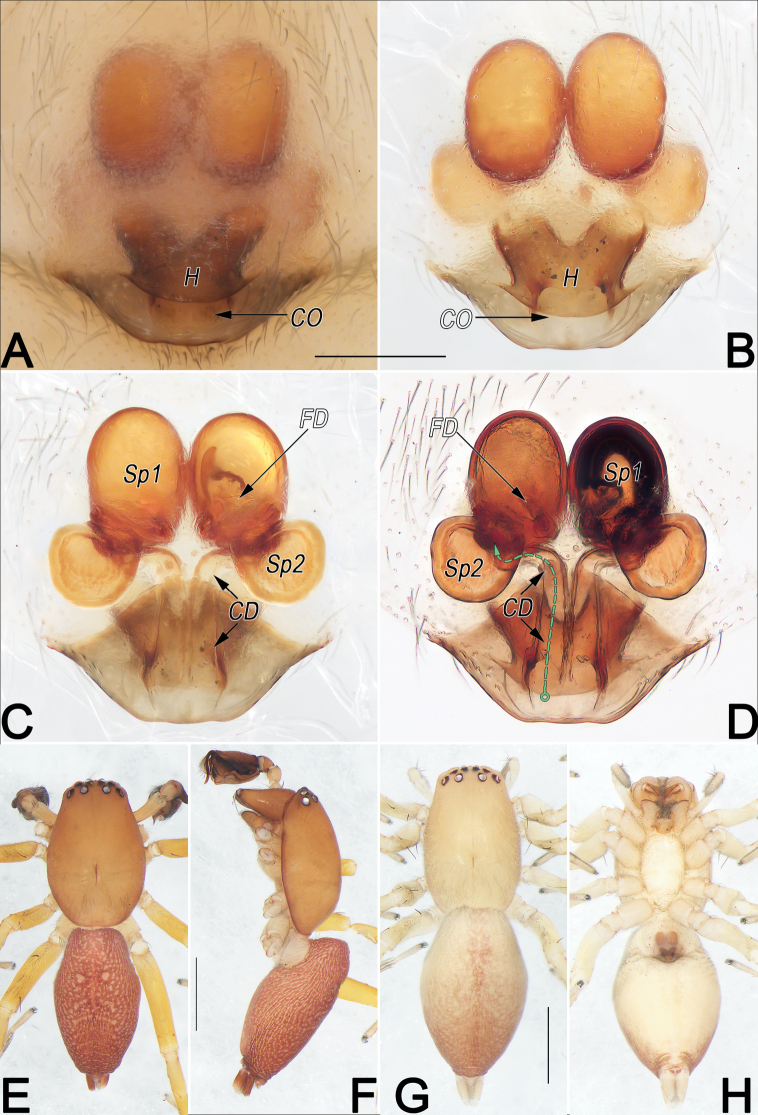
*Clubionanezha* sp. nov., female paratype and male holotype, epigyne (A–D), male habitus (E, F) and female habitus (G, H). A. Intact, ventral view; B. Cleared, ventral view; C. Cleared, dorsal view; D. Cleared, dorsal view (dashed line in D showing schematic course of copulatory duct, dorsal); E. Dorsal view; F. Lateral view; G. Dorsal view; H. Ventral view. Abbreviations: CD = copulatory duct; CO = copulatory opening; FD = fertilisation duct; H = hood; Sp1 = primary spermatheca; Sp2 = secondary spermatheca. Scale bars: 0.2 mm (A–D); 1 mm (E–H).

*Clubionanezha* sp. nov. resembles *C.jiugong* Yu & Zhong, 2021 but differs in the following: (1) tegulum (Te) shaped like inverted triangle, ~1.3× longer than wide (vs oval, ~2× as long as wide) (cf. Fig. 12D and Zeng et al. 2021: figs 1C, 2D); (2) process of the embolic base (EBP) slightly longer than tegulum width, tip slightly overpasses retrolateral rim of tegulum (vs slightly shorter than tegulum width, tip does not reach retrolateral rim of tegulum) (cf. Fig. 12D and Zeng et al. 2021: figs 1C, 2D); (3) course of copulatory ducts (CD) shaped like the number 7 (vs shaped like the symbol >) (cf. Fig. 13C, D and Zeng et al. 2021: fig. 3C, D).

##### Description.

**Male.** Holotype (Fig. 13E, F): Total length 4.25; carapace 2.01 long, 1.34 wide; abdomen 2.24 long, 1.19 wide. Carapace uniformly reddish-orange, without distinct pattern; cephalic region distinctly narrowed, cervical groove and radial grooves indistinct; tegument smooth, laterally and posteriorly clothed with short, fine setae. Eyes: both AER and PER slightly recurved in dorsal view, latter wider than former. Eye sizes and interdistances: AME 0.08, ALE 0.13, PME 0.12, PLE 0.12, AME–AME 0.09, AME–ALE 0.05, PME–PME 0.21, PME–PLE 0.13, MOQL 0.28, MOQA 0.25, MOQP 0.42. Chelicerae robust, coloured as carapace, both margins with five teeth. Sternum pale brown, 1.01 long, 0.67 wide. Labium and endites pale orange. Legs uniformly bright yellow, without distinct markings. Leg measurements: I 3.72 (1.06, 1.56, 0.73, 0.37), II 4.07 (1.19, 1.67, 0.81, 0.40), III 3.45 (1.05, 1.18, 0.82, 0.40), IV 5.25 (1.57, 1.79, 1.39, 0.50). Abdomen elongate-oval, dorsum basically reddish brown, mottled with countless small beige spots and stripes: anteriorly with pair of circular muscular depressions at the front 1/3, posteriorly with five or six indistinct transverse chevrons; venter basically pale peach, with four longitudinal dotted lines.

Palp (Fig. 12A–E). Tibia short, ~1/4 of cymbium (Cy) length, with single retrolateral apophysis (RTA); RTA ~1.2× longer than tibia, broad, triangular in retrolateral view, apex slightly bifurcate, both tips blunt. Subtegulum (ST) large, > 5/6 of tegulum length, prolateral surface membranous, wrinkled, ribbed, with incomputable diagonal ridges. Tegulum (Te) inverted triangle-shaped, ~1.3× longer than wide, sperm duct (SD) distinct and sinuous. embolic base (EB) situated ~9–11 o’clock position; embolic base process (EBP) slightly longer than tegulum width, dagger-shaped, originating on prolateral flank (~11 o’clock), transversally curved to retrolateral side, terminating at ~1 o’clock position, tip slightly overpasses retrolateral rim of tegulum. Embolus (Em) slender, flagelliform, bent on right angle, stretched proximally along tegular groove, tip extending to 1/3 of tegulum length. Tegular groove (TG) relatively small, ~2/5 of tegulum length.

**Female.** Paratype (Fig. 13G, H): Total length 4.23; carapace 1.93 long, 1.27 wide; abdomen 2.30 long, 1.30 wide. Eye sizes and interdistances: AME 0.09, ALE 0.11, PME 0.11, PLE 0.10, AME–AME 0.08, AME–ALE 0.04, PME–PME 0.21, PME–PLE 0.13, MOQL 0.26, MOQA 0.24, MOQP 0.42. Sternum 1.02 long, 0.59 wide. Leg measurements: I 3.25 (0.94, 1.39, 0.62, 0.30), II 3.46 (1.01, 1.46, 0.69, 0.30), III 3.10 (0.91, 1.09, 0.79, 0.31), IV 4.97 (1.50, 1.73, 1.27, 0.47). Similar to male but distinctly larger and paler.

Epigyne (Fig. 13A–D). Epigynal plate ~1.1× longer than wide, anterior and lateral margins not delimited, posterior margin rebordered, strongly sclerotised, convex; spermathecae clearly visible through tegument. Hood (H) V-shaped, located posteriorly, ~1/3 of epigyne length and 3/5 of epigyne width, translucent, through which anterior parts of copulatory openings easily visible. Copulatory openings (CO) oval, large, ~1/6 of epigyne length and 1/8 of epigyne width, partly fused along middle line, situated medial portion of posterior margin of epigynal plate, anteriorly hidden by hood. Hyaline copulatory ducts (CD) thin, running in parallel, first 3/5s juxtaposed vertically, ascending to ~1/2 length of endogyne, then bend ~90°, extending laterally, finally entering secondary spermathecae (Sp2) and curving slightly anteriorly, nearly forming a 7-shaped course. Both primary (Sp1) and secondary spermathecae (Sp2) with smooth surfaces, former situated anteriorly and distinctly larger than latter. Primary spermathecae closely spaced, egg-shaped, large, ~2/5 of epigyne length. Secondary spermathecae globular, separated by ~1× diameter. Fertilisation ducts (FD) located on basal-mesal surface of primary spermathecae.

##### Distribution.

Known only from the type locality, Jiangyou County-level City, Sichuan, China (Fig. 1).

##### Etymology.

The species is named after Nezha, the youthful hero deity in ancient Chinese mythology, noun in apposition. Nezha is often depicted wearing a red battle robe (the body color of the holotype of *C.nezha* sp. nov. is also predominantly red).

## Supplementary Material

XML Treatment for Clubiona

XML Treatment for Clubiona huntianling

XML Treatment for Clubiona rouqiu

XML Treatment for Clubiona yinyangjian

XML Treatment for Clubiona huojianqiang

XML Treatment for Clubiona qiankunquan

XML Treatment for Clubiona nezha

## ﻿Additional information

### Use of AI

No use of AI was reported.

### Author ORCIDs

Jianshuang Zhang https://orcid.org/0000-0003-4010-3082

Hao Yu https://orcid.org/0000-0002-9113-2425

Shuqiang Li https://orcid.org/0000-0002-3290-5416

### Data availability

All of the data that support the findings of this study are available in the main text or Supplementary Information.

## Supplementary materials

Supplementary material 1 The comparison of diagnostic illustration between the six new species and their similar species Data type pdf https://binary.pensoft.net/file/1387739This dataset is made available under the Open Database License (http://opendatacommons.org/licenses/odbl/1.0/). The Open Database License (ODbL) is a license agreement intended to allow users to freely share, modify, and use this Dataset while maintaining this same freedom for others, provided that the original source and author(s) are credited.Hao Yu
