# Supplementary material for: ﻿Six new species of the spider genus Clubiona Latreille, 1804 (Araneae, Clubionidae) from subtropical forests of Sichuan Province, China
Source: Zookeys. 2025 Aug 4;1248:61–91. doi: 10.3897/zookeys.1248.153967 (PMC12340537; doi:10.3897/zookeys.1248.153967)

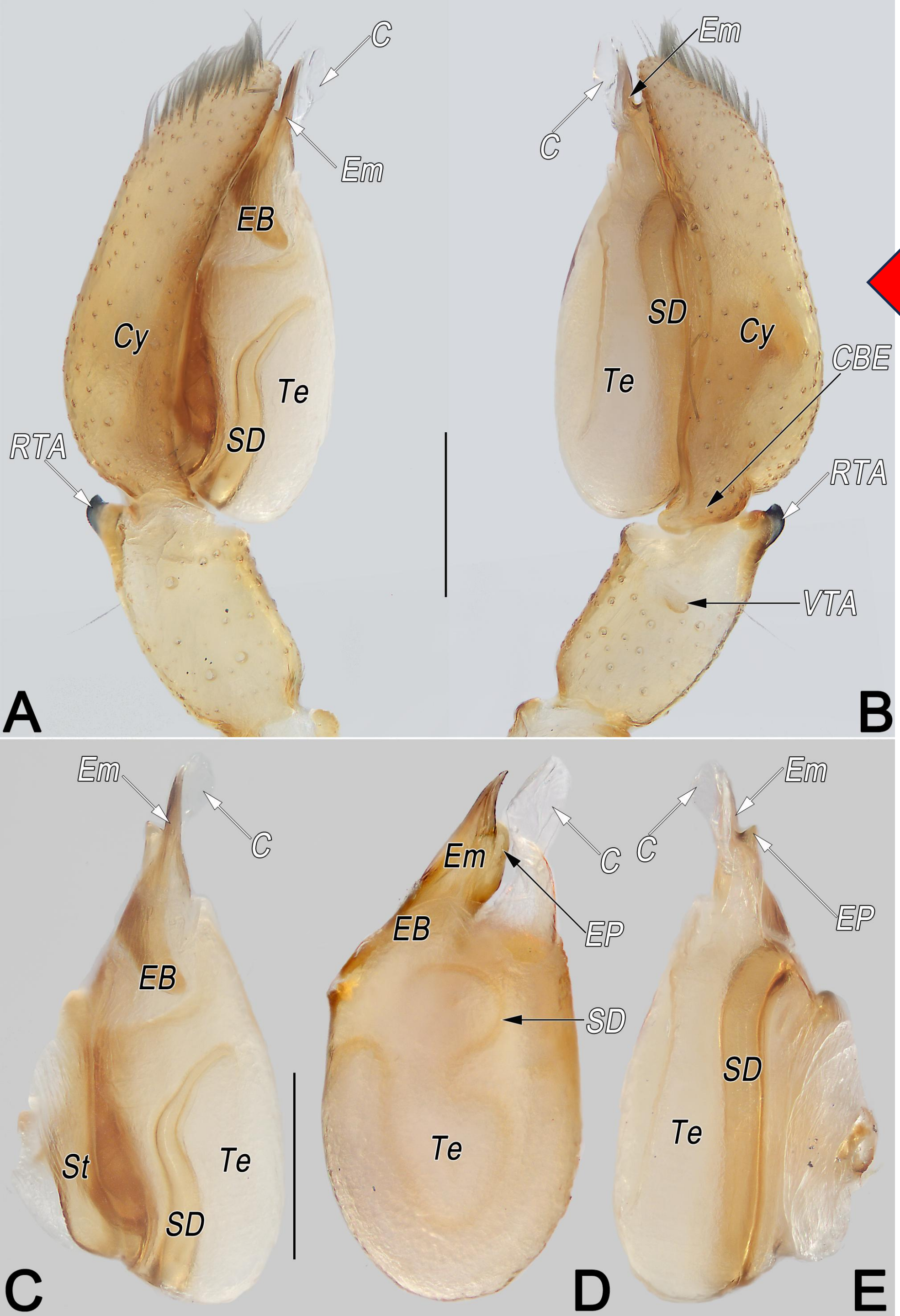

C. huntianling sp. nov. (left)
vs.
C. pianmaensis (right)

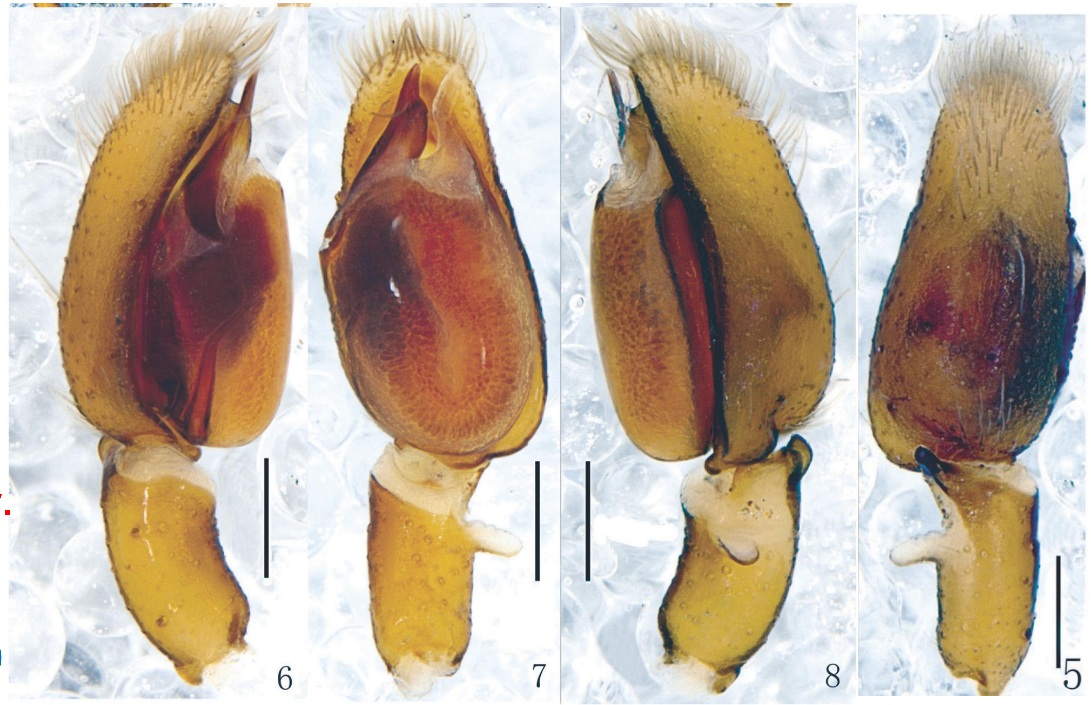

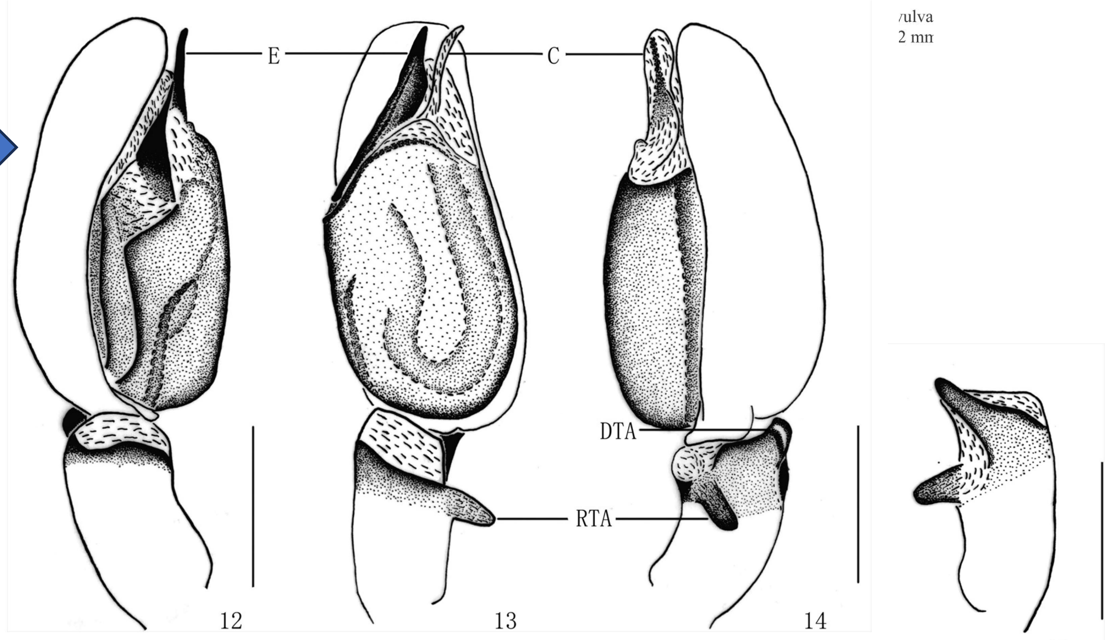

Figs. 9–14. *Clubiona pianmaensis* sp. nov. 9, epigyne, ventral view; 10, vulva; 11, retrolateral tibial apophysis, dorsal view; 12, mal palp, prolateral view; 13, same, ventral view; 14, same, retrolateral view. Scales = 0.2 mm.

C. huntianling sp. nov. (left) vs. *C. pianmaensis* (right)

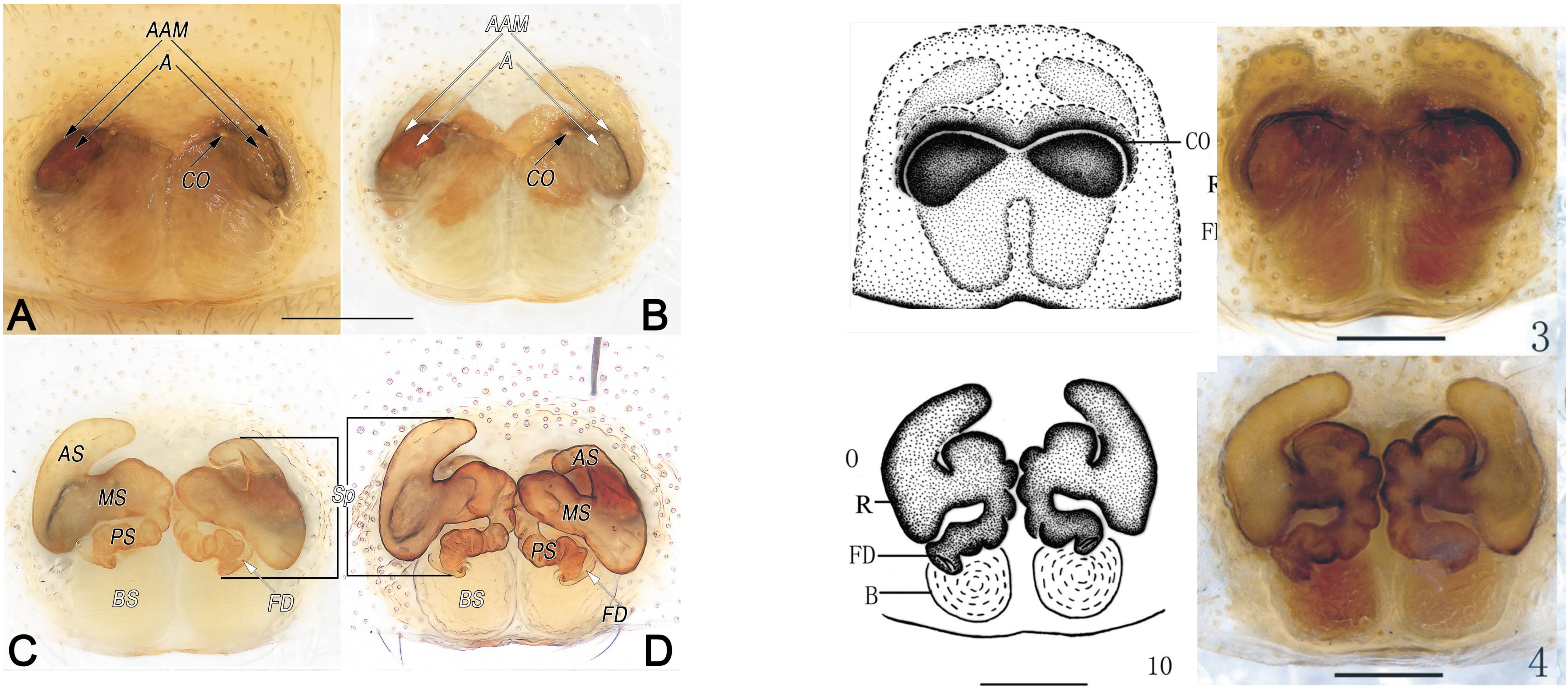

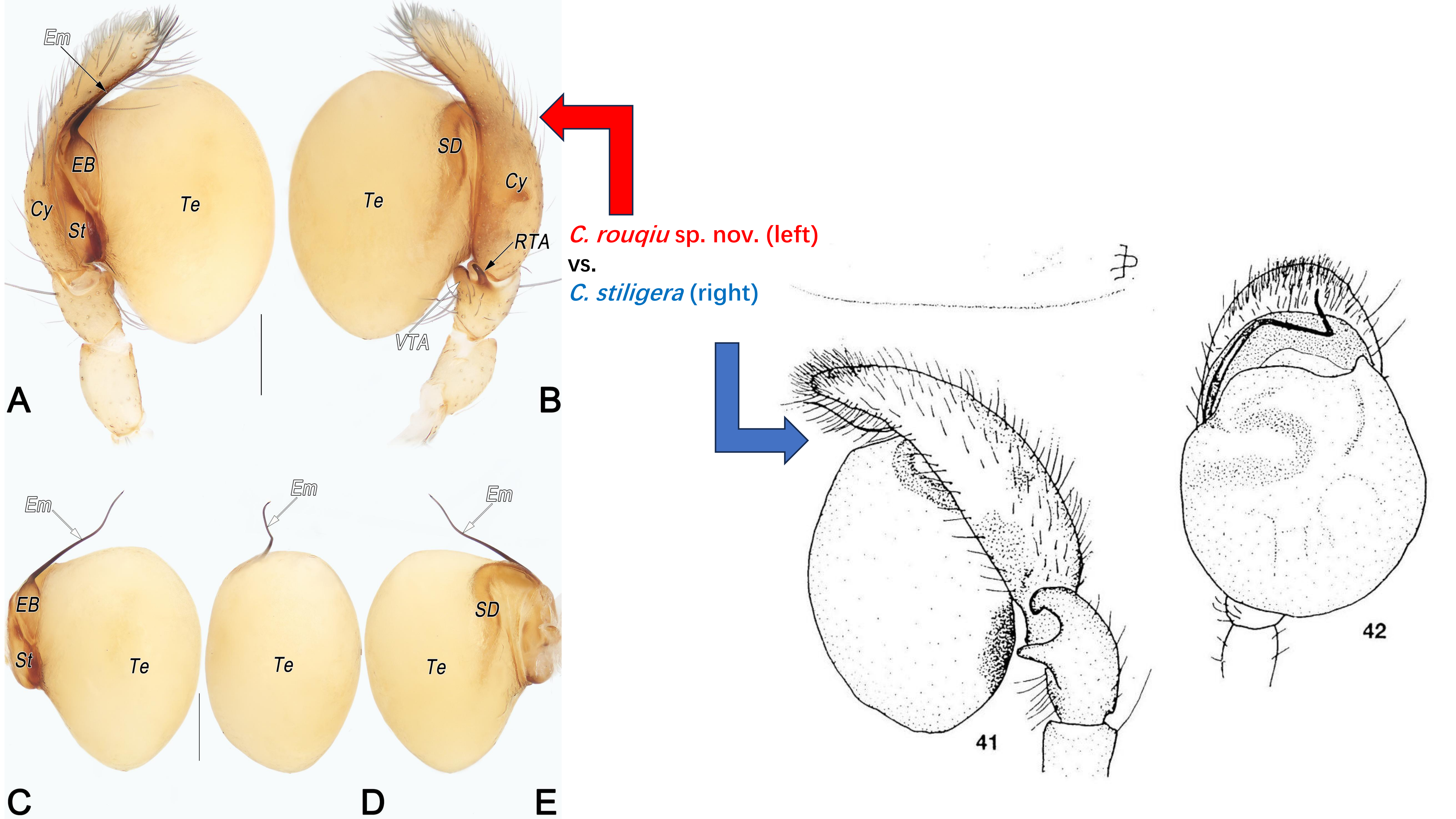
Em
EB
Cy
St
Te
A
SD
Te
Cy
RTA
VTA
B
C. rouqiu sp. nov. (left)
vs.
C. stiligera (right)
41
42
Em
EB
St
Te
C
Em
Te
D
Em
SD
Te
E

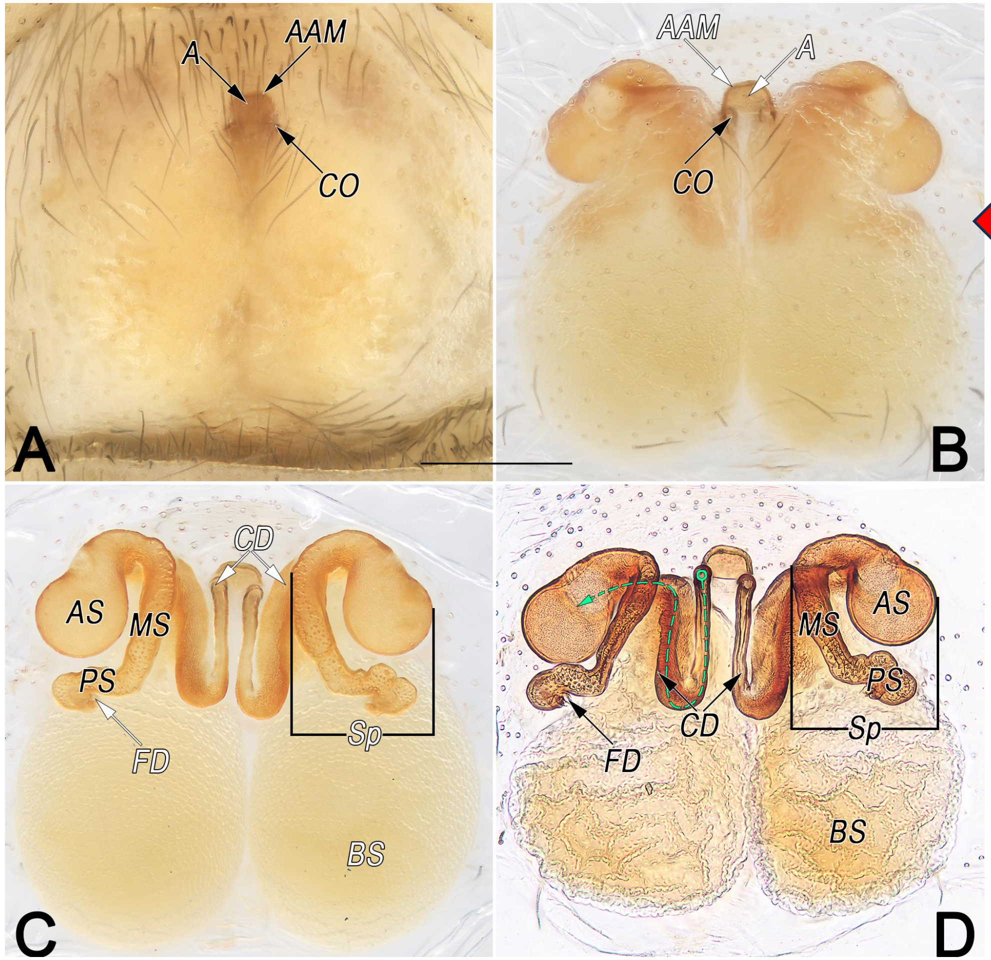

C. rouqiu sp. nov. (left)
vs.
C. tiane (right)

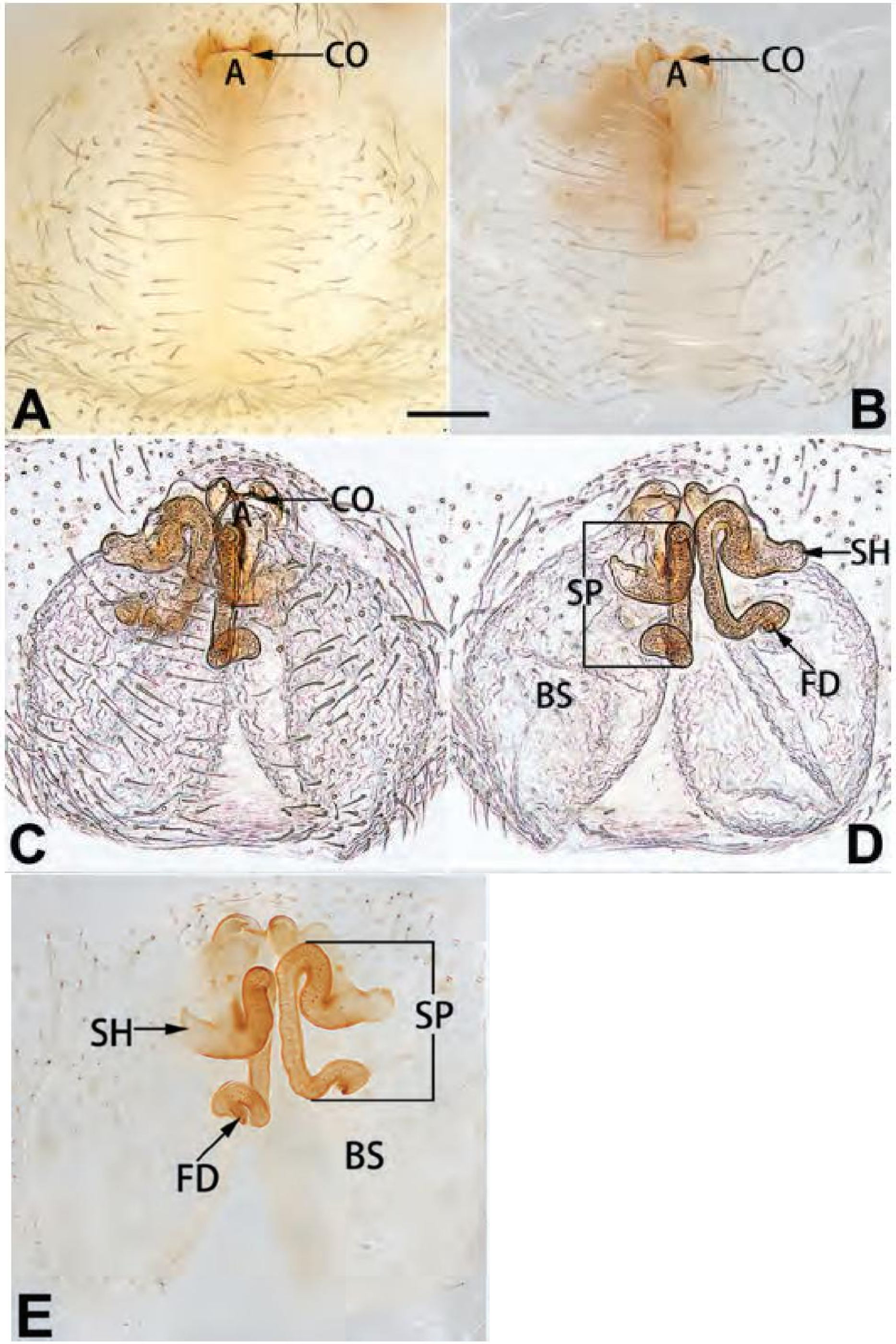

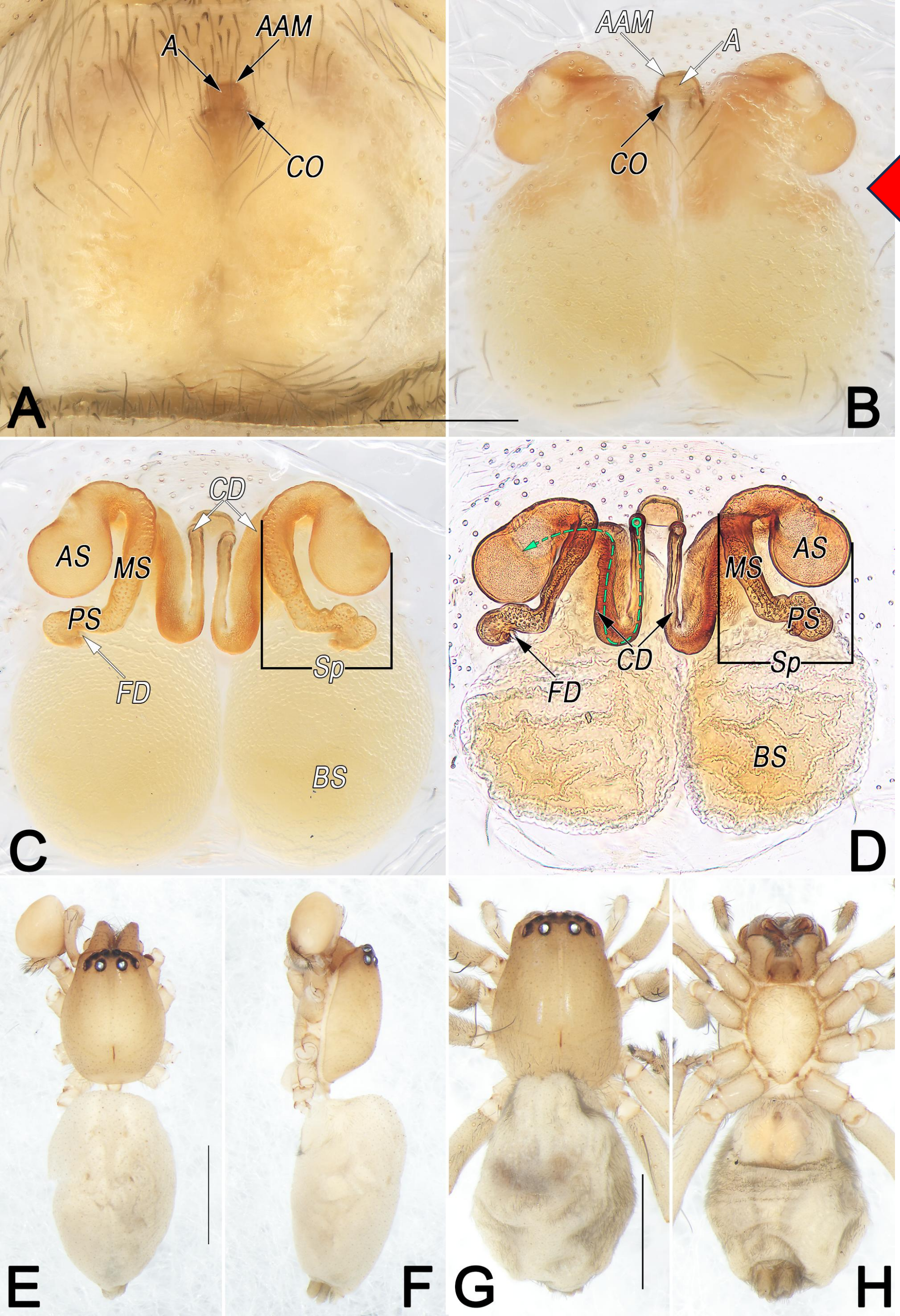

C. yinyangjian sp. nov. (left)
vs.
C. tengchong (right)

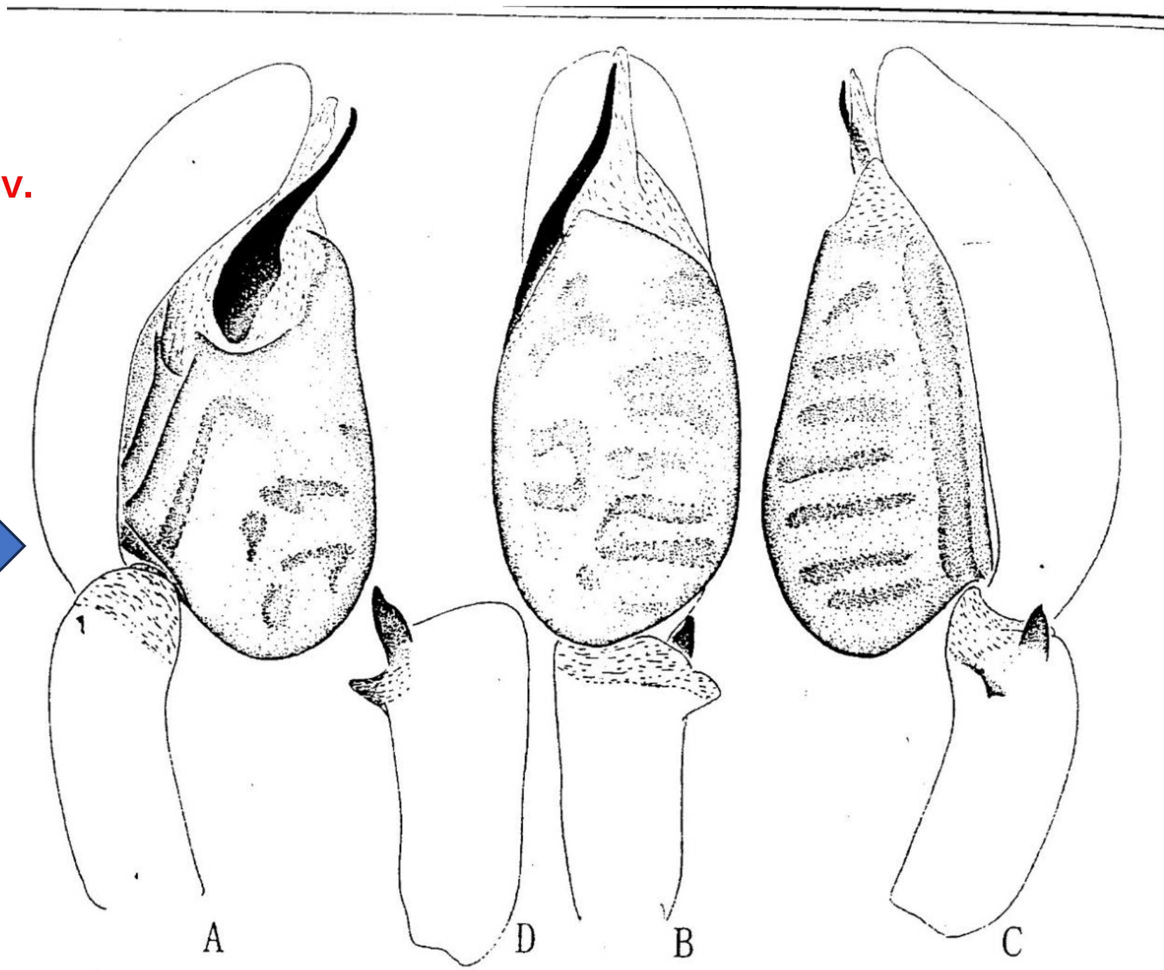

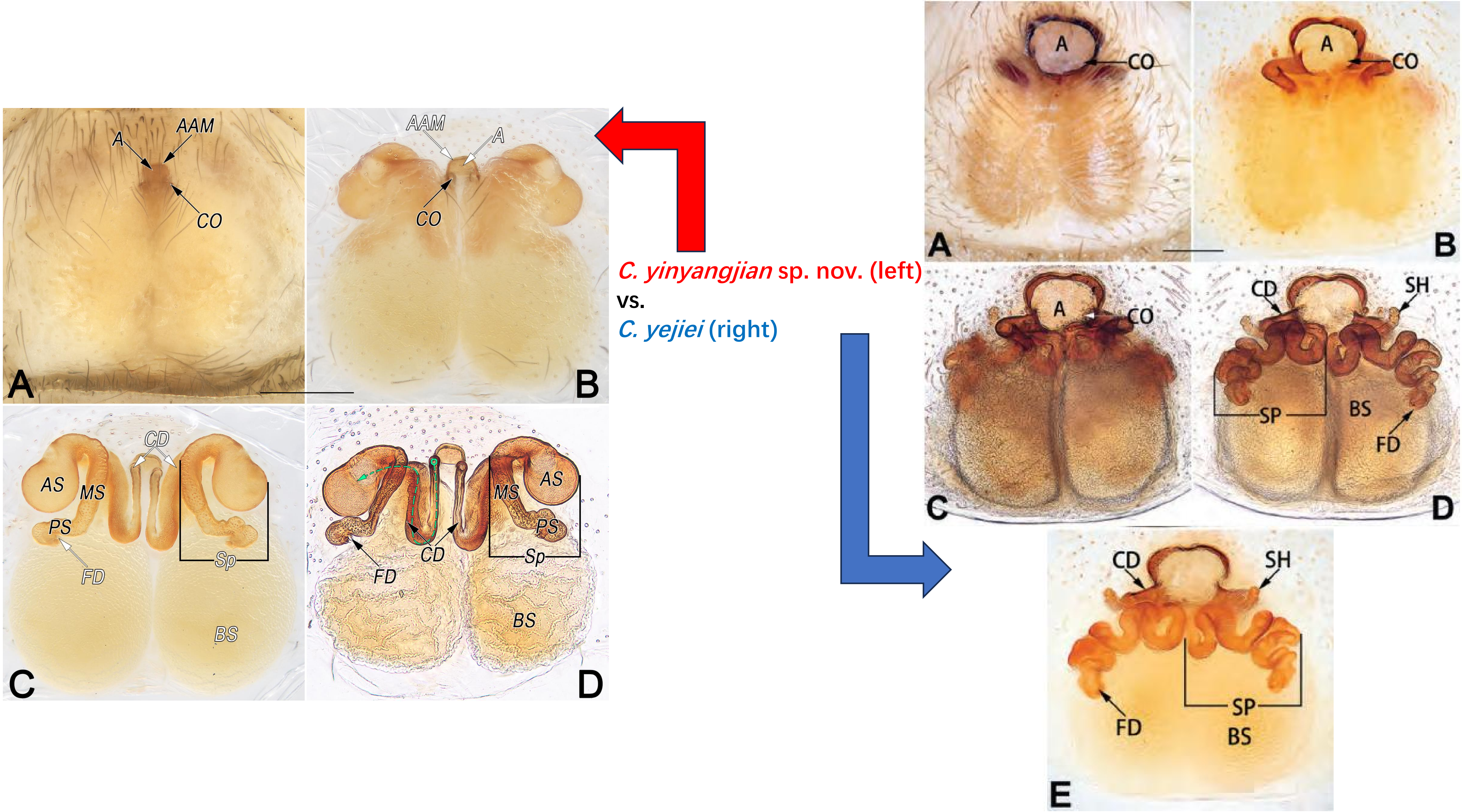

C. yinyangjian sp. nov. (left)
vs.
C. yejiei (right)
A
AAM
CO
B
CD
AS
MS
PS
FD
Sp
BS
C
D
SH
SP
E

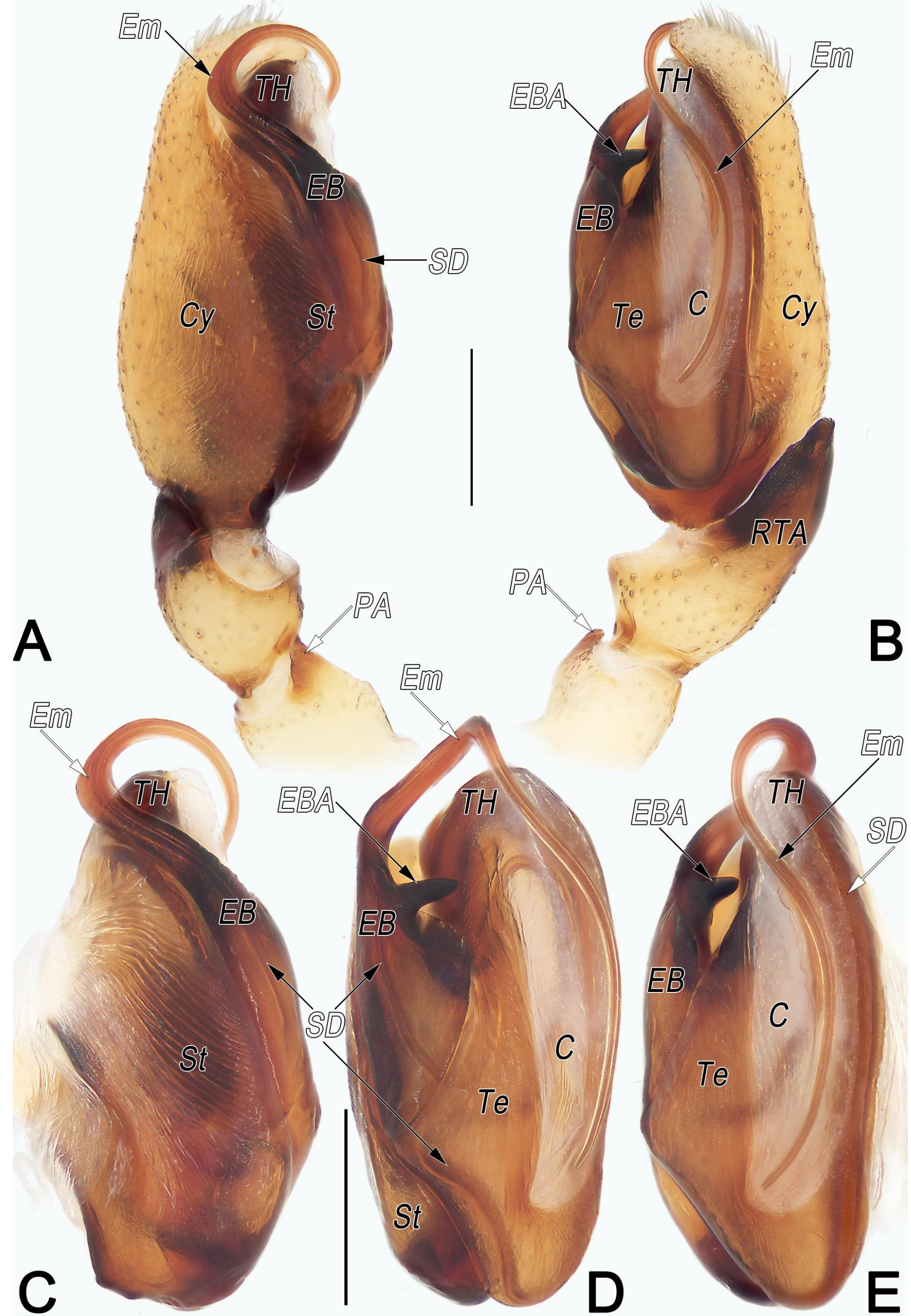

C. huojianqiang sp. nov. (left)

vs.

C. contrita (right)

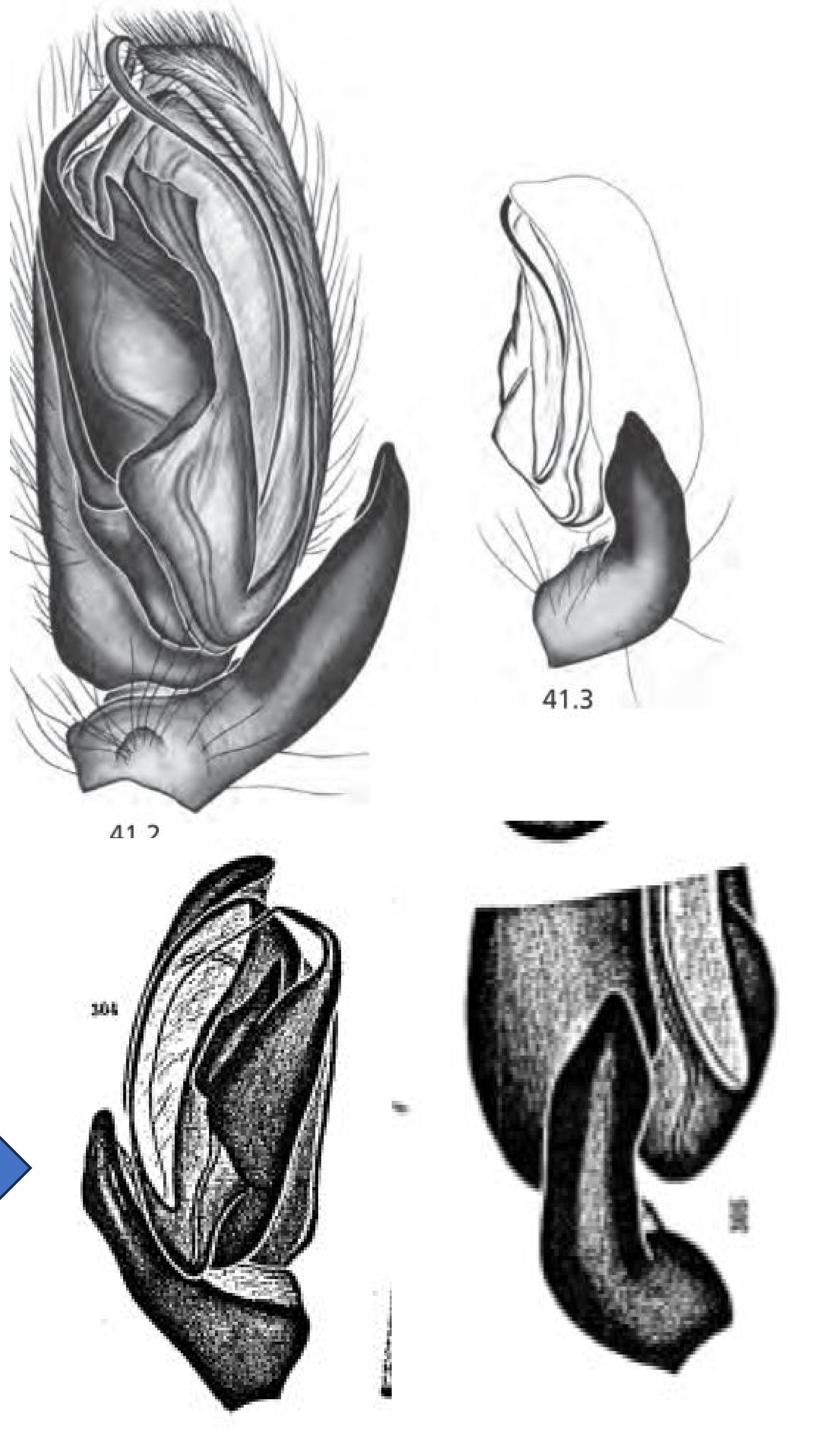

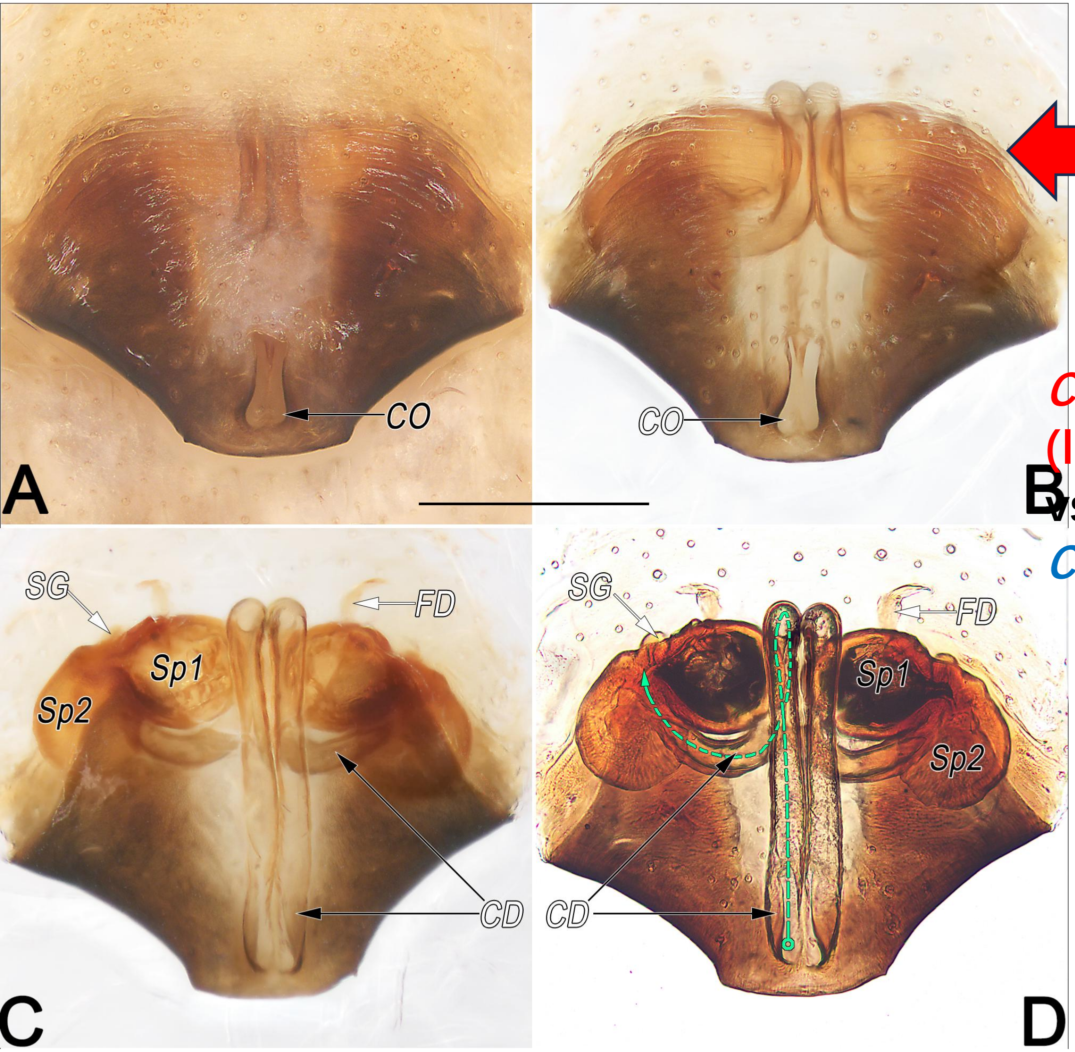

C. huojianqiang sp. nov. (left)
vs.
C. contrita (right)

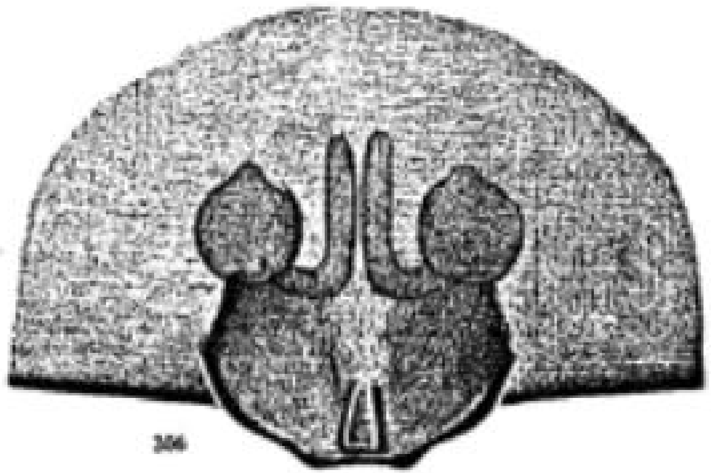

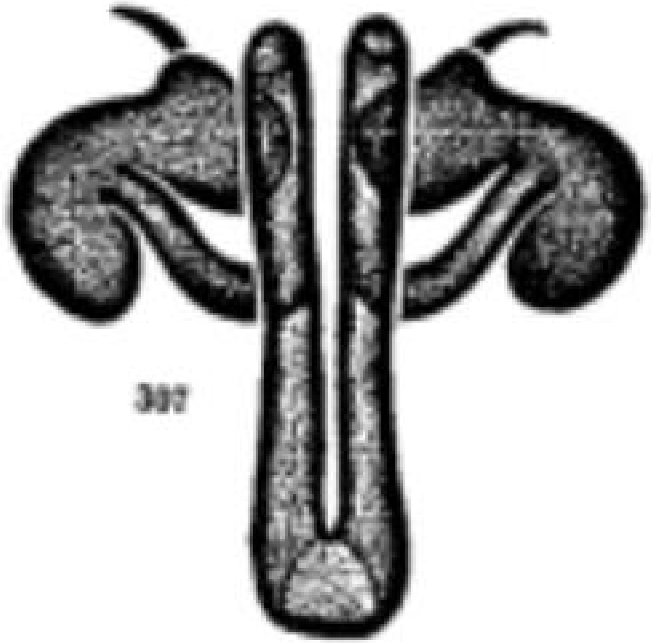

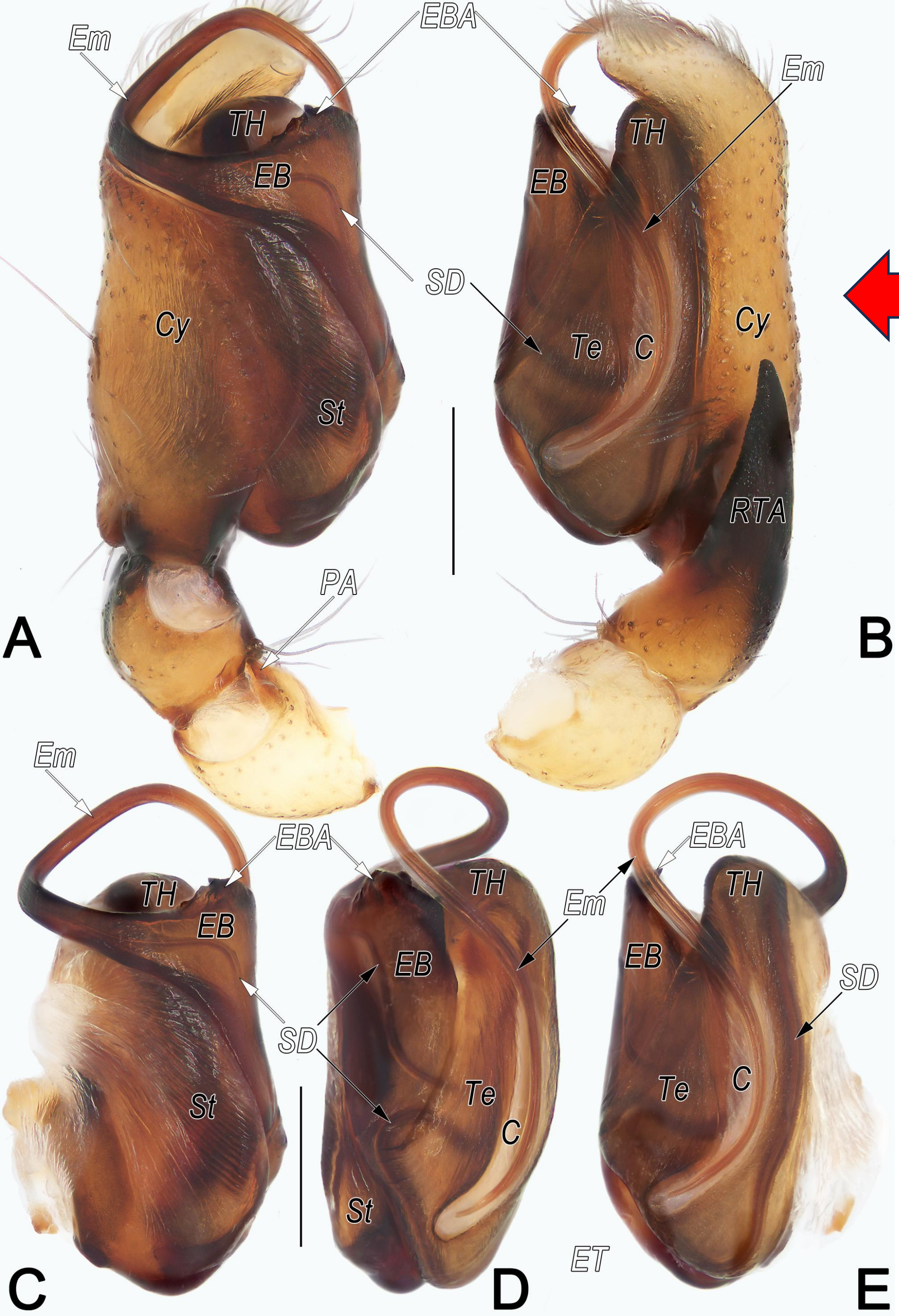

C. qiankunquan sp. nov. (left)
vs.
C. subasrevida (right)

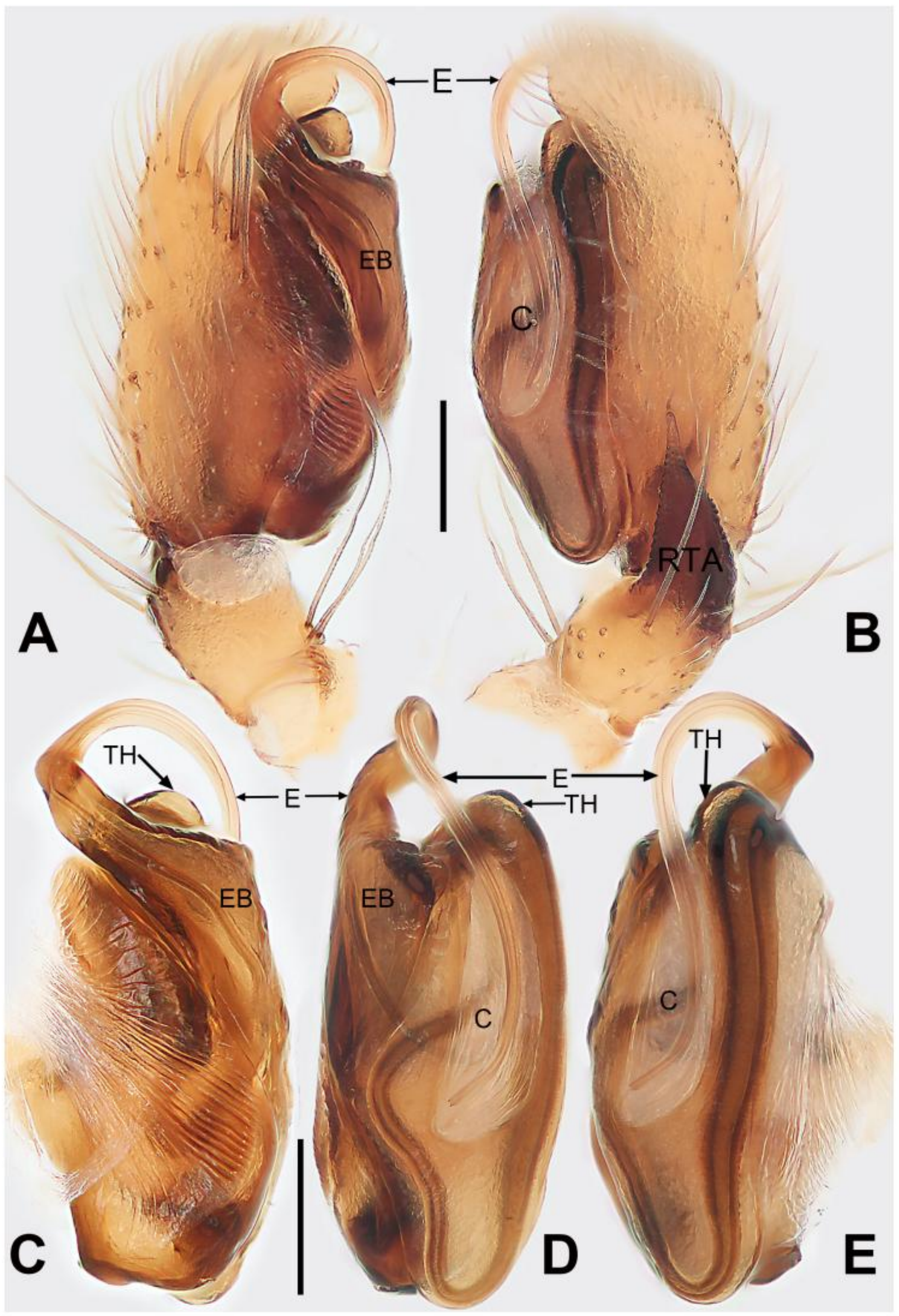

FIGURE 17. *Clubiona subasrevida* **sp. nov.**, male holotype: A–B, left palp (A prolateral view; B retrolateral view); C–E, left palpal bulb (C prolateral view; D ventral view; E retrolateral view). Abbreviatures: C, conductor; E, embolus; EB, embolic base; RTA, retrolateral tibial apophysis; TH, tegular hump. Scale bars: 0.10 mm (A–B, C–E in the same scale).

C. qiankunquan sp. nov. (left) vs. *C. subasrevida* (right)

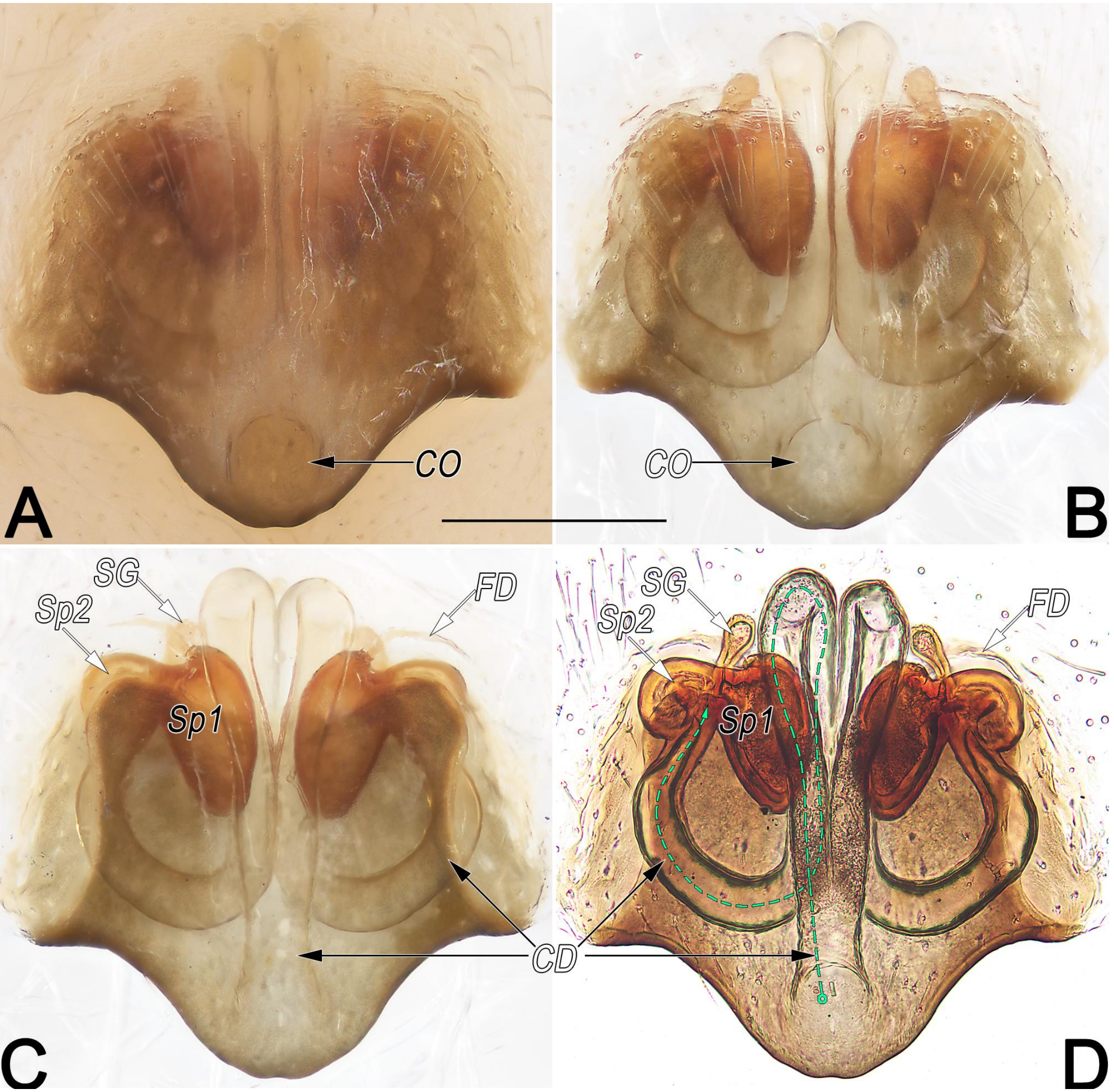

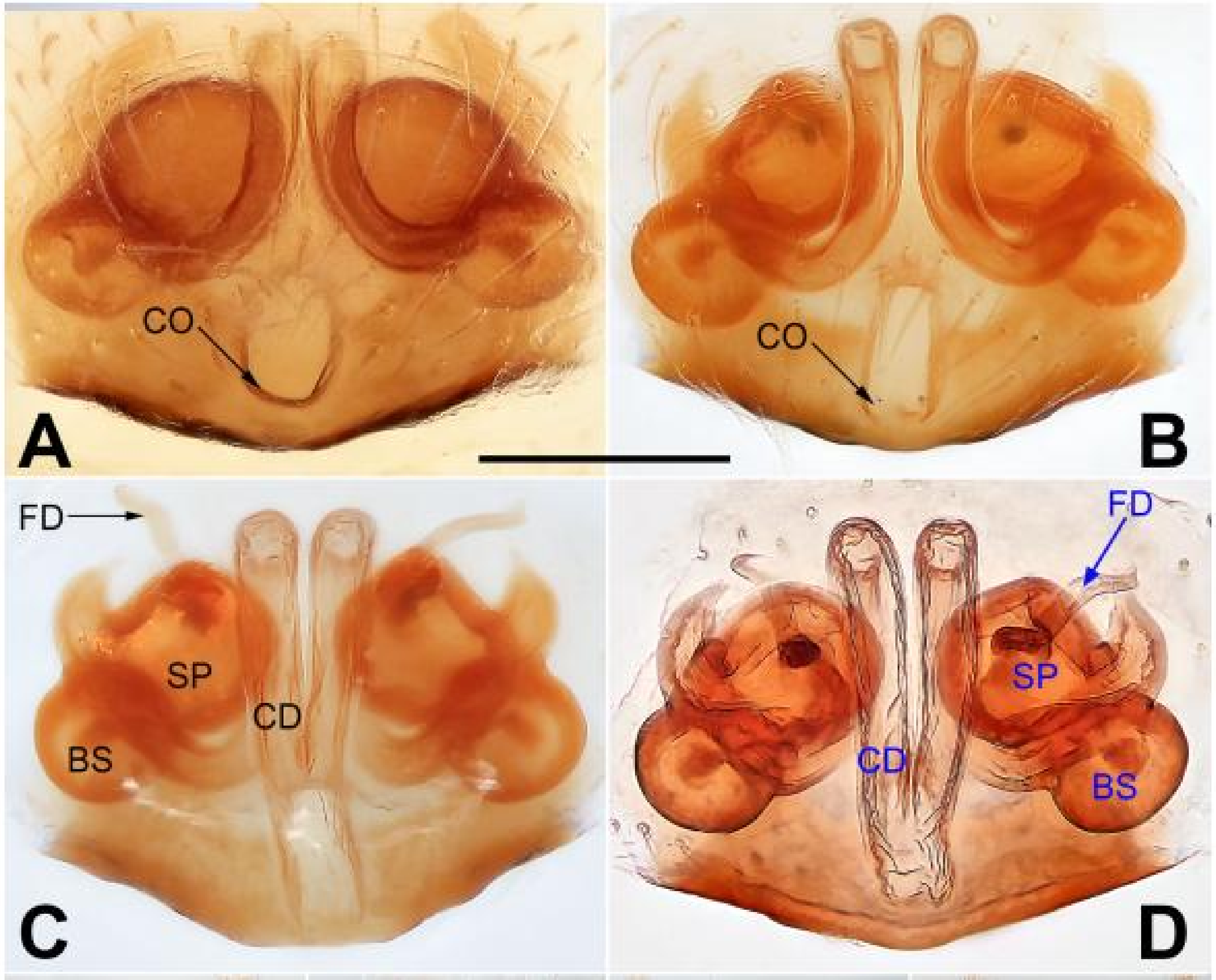

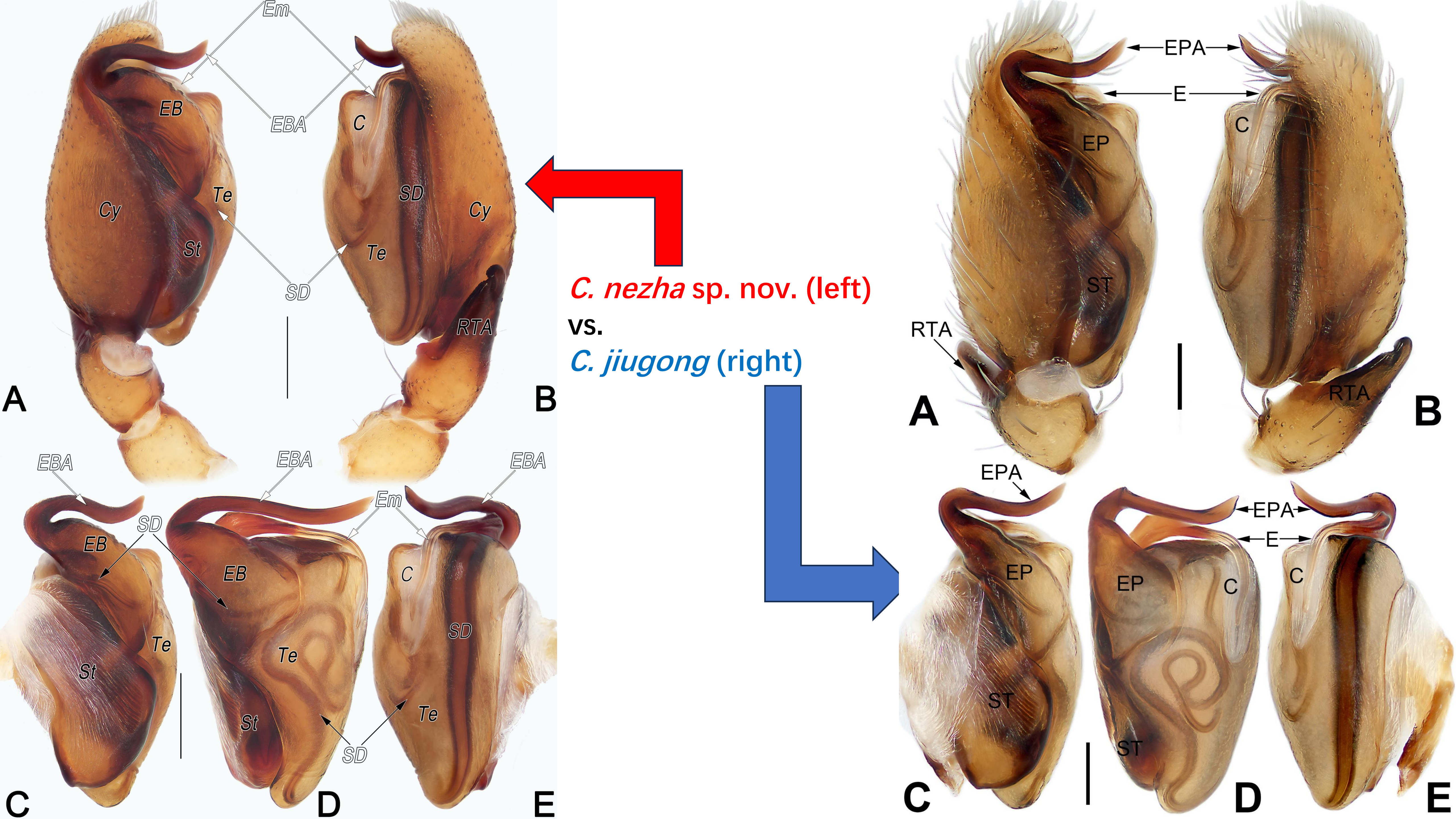

C. nezha sp. nov. (left)
vs.
C. jiugong (right)
Em
EB
EBA
C
SD
Te
Cy
St
RTA
A
B
C
D
E
EPA
E
EP
ST

C. nezha sp. nov. (left) vs. *C. jiugong* (right)

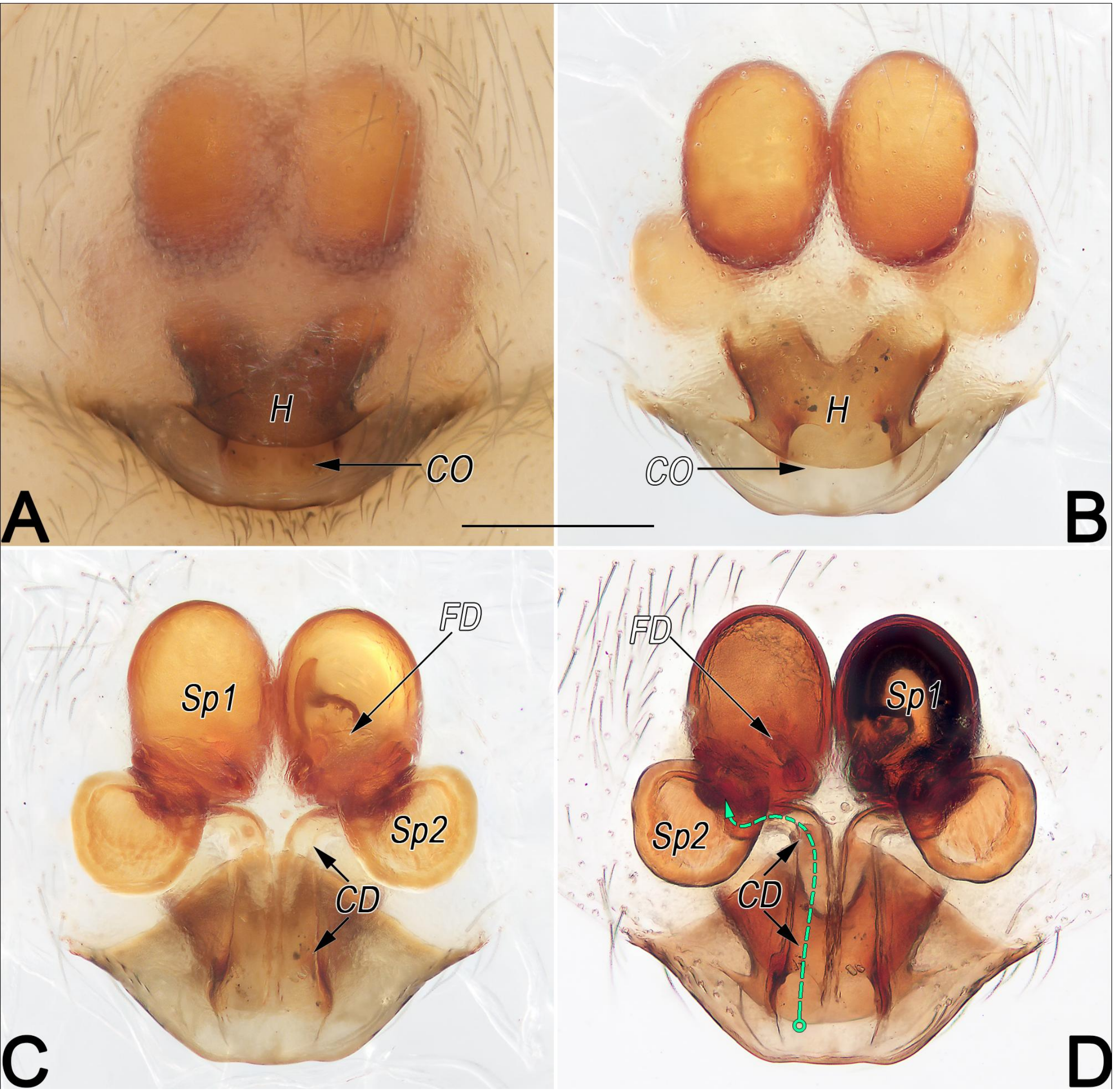

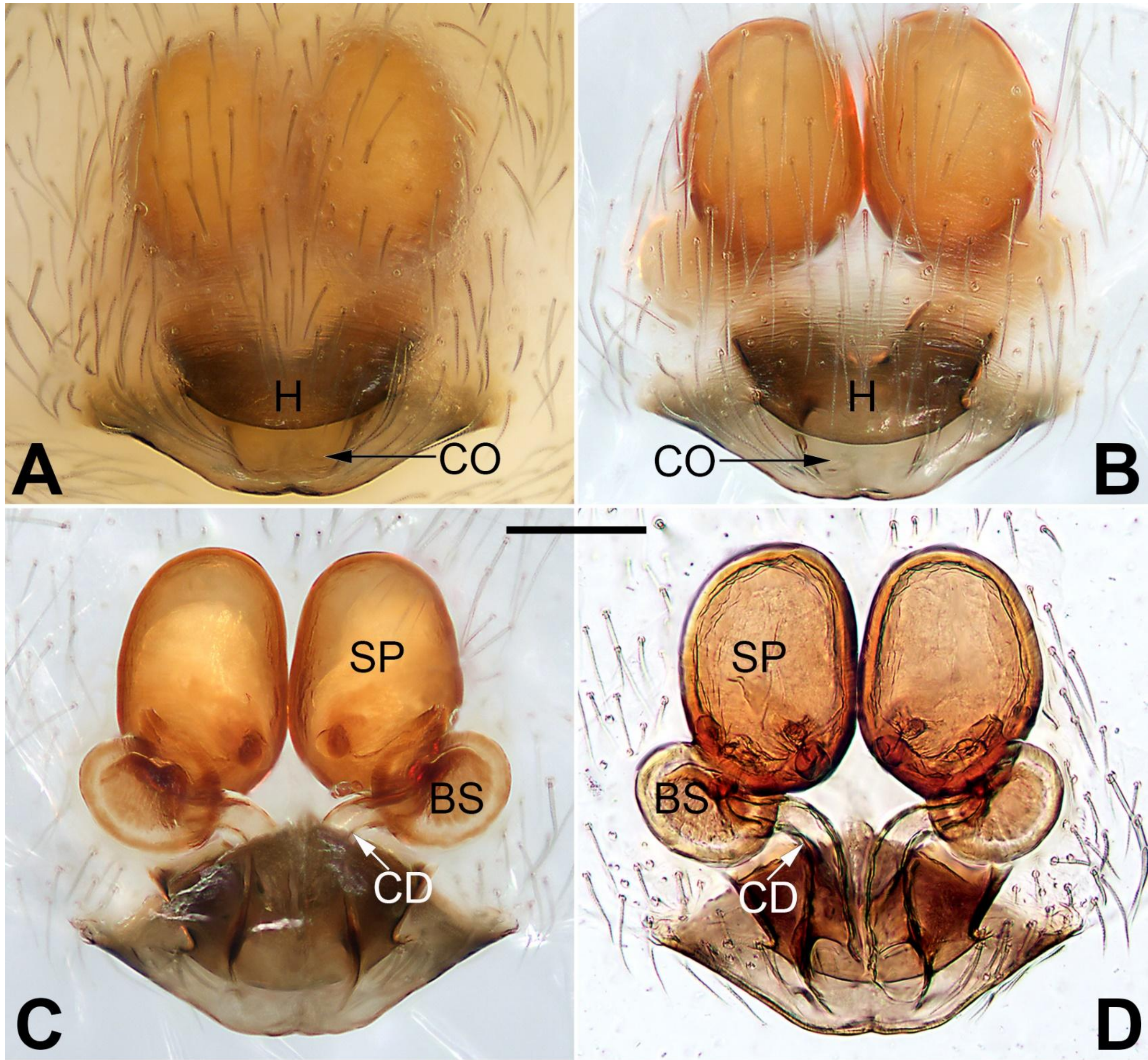

Supplement: Supplementary material 1 — The comparison of diagnostic illustration between the six new species and their similar species [file zookeys-1248-061_article-153967__-s001.pdf]
